# Genome-wide analysis of DNA methylation in buccal cells: a study of monozygotic twins and mQTLs

**DOI:** 10.1186/s13072-018-0225-x

**Published:** 2018-09-25

**Authors:** Jenny van Dongen, Erik A. Ehli, Rick Jansen, Catharina E. M. van Beijsterveldt, Gonneke Willemsen, Jouke J. Hottenga, Noah A. Kallsen, Shanna A. Peyton, Charles E. Breeze, Cornelis Kluft, Bastiaan T. Heijmans, Meike Bartels, Gareth E. Davies, Dorret I. Boomsma

**Affiliations:** 1 Department of Biological Psychology, Amsterdam Public Health Research Institute, Vrije Universiteit Amsterdam, Van Der Boechorststraat 1, 1081BT Amsterdam, The Netherlands; 2 Avera Institute for Human Genetics, 3720 W. 69th Street, Sioux Falls, SD 57108 USA; 3 Department of Psychiatry, VU University Medical Center, Oldenaller 1, 1081 HJ Amsterdam, The Netherlands; 4 Altius Institute for Biomedical Sciences, 2211 Elliott Ave, Seattle, WA 98121 USA; 5 Good Biomarker Sciences, Zernikedreef 8, 2333 CL Leiden, The Netherlands; 6 Molecular Epidemiology Section, Leiden University Medical Center, Postal Zone S-05-P, PO Box 9600, 2300 RC Leiden, The Netherlands

**Keywords:** DNA methylation, Epigenetics, Illumina, 450 k, EPIC, Array, Twin study, Buccal, Children, QTL

## Abstract

**Background:**

DNA methylation arrays are widely used in epigenome-wide association studies and methylation quantitative trait locus (mQTL) studies. Here, we performed the first genome-wide analysis of monozygotic (MZ) twin correlations and mQTLs on data obtained with the Illumina MethylationEPIC BeadChip (EPIC array) and compared the performance of the EPIC array to the Illumina HumanMethylation450 BeadChip (HM450 array) for buccal-derived DNA.

**Results:**

Good-quality EPIC data were obtained for 102 buccal-derived DNA samples from 49 MZ twin pairs (mean age = 7.5 years, range = 1–10). Differences between MZ twins in the cellular content of buccal swabs were a major driver for differences in their DNA methylation profiles, highlighting the importance to adjust for cellular composition in DNA methylation studies of buccal-derived DNA. After adjusting for cellular composition, the genome-wide mean correlation (*r*) between MZ twins was 0.21 for the EPIC array, and *cis* mQTL analysis in 84 twins identified 1,296,323 significant associations (FDR 5%), encompassing 33,749 methylation sites and 616,029 genetic variants. MZ twin correlations were slightly larger (*p* < 2.2 × 10^−16^) for novel EPIC probes (*N* = 383,066, mean *r* = 0.22) compared to probes that are also present on HM450 (*N* = 406,822, mean *r* = 0.20). In line with this observation, a larger percentage of novel EPIC probes was associated with genetic variants (novel EPIC probes with significant mQTL 4.7%, HM450 probes with mQTL 3.9%, *p* < 2.2 × 10^−16^). Methylation sites with a large MZ correlation and sites associated with mQTLs were most strongly enriched in epithelial cell DNase I hypersensitive sites (DHSs), enhancers, and histone mark H3K4me3.

**Conclusions:**

We conclude that the contribution of familial factors to individual differences in DNA methylation and the effect of mQTLs are larger for novel EPIC probes, especially those within regulatory elements connected to active regions specific to the investigated tissue.

**Electronic supplementary material:**

The online version of this article (10.1186/s13072-018-0225-x) contains supplementary material, which is available to authorized users.

## Background

The Illumina HumanMethylation450 BeadChip (HM450 array) [1], which measures DNA methylation at approximately 485,000 methylation sites (mostly CpG sites), has been widely used to measure genome-wide DNA methylation and was recently replaced by the MethylationEPIC BeadChip (EPIC array) [2], which measures DNA methylation at > 850,000 methylation sites (including ~ 90% of sites from the HM450 array). Several validation studies of the EPIC array have been published that assessed the reproducibility of the EPIC array, compared the performance of the EPIC array to the HM450 array, or compared the performance of the EPIC array to whole-genome bisulfite sequencing (WGBS) [2–5]. These studies have reported high correlations (*r* > 0.9, across all CpGs) between replicate samples on EPIC and between matched samples measured on HM450 and EPIC (*r* > 0.9, across all overlapping CpGs). A study of whole blood indicated that correlations for many individual CpGs are fairly low between HM450 and EPIC (*r* < 0.2 at 55% of CpGs) [5], due to the low variance of methylation levels of most CpGs. However, replication of trait-associated CpGs across the HM450 and EPIC arrays has been reported for cancer-associated differential methylation [3], CpGs associated with maternal smoking [6], C-reactive protein (CRP) [5], and the epigenetic clock [5]. Validation studies have been performed for DNA derived from a variety of different samples, including primary normal colon [2], primary sorted neurons [2], renal cancer [2], a transformed prostate cancer cell line [3], primary cultures of prostate epithelial cells [3], cancer-associated fibroblasts and non-malignant tissue-associated fibroblasts [3], pediatric brain tumors [4], infant blood from Guthrie cards [3], and whole blood [5]. Thus far, no study has been published on EPIC data generated with DNA derived from buccal swabs, which may be used as a surrogate tissue in epigenome-wide association studies of human traits and in studies of genetic variants that influence DNA methylation.

Methylome-wide studies in monozygotic (MZ) and dizygotic (DZ) twins are performed to obtain insight into the extent to which DNA methylation levels are influenced by genetic, environmental and stochastic influences or to identify loci where methylation differences between twins are associated with discordance for traits [7]. MZ (identical) twins have nearly identical DNA sequences, although they may differ with respect to post-zygotic somatic mutations [8–10]. Their DNA methylation profiles show differences in multiple tissues that are already detectable at birth, and these difference may increase with age [11–14]. We, and others, have previously used the HM450 array to assess genome-wide DNA methylation in buccal swabs from twins [15, 16]. In our previous study, we assessed DNA methylation in buccal swabs from ten monozygotic (MZ) pairs (age 8–19) and found that the correlation between methylation values of MZ twins at individual CpGs varies across the genome, with a mean across all CpGs on the HM450 array of 0.31 [16]. Correlations between MZ twins provide an indication of the relative importance of familial factors (genetic variation and shared environment) versus the importance of environmental and stochastic influences to inter-individual variation in methylation levels. Previous methylation QTL (mQTL) studies of tissues including whole blood, adipose, lung, and brain have shown that methylation sites interrogated by the HM450 array show widespread associations with common genetic variants [17–20]. To our knowledge, such studies have not yet been performed for buccal-derived DNA or for the EPIC array.

In the current study, we measured DNA methylation with the EPIC array in 107 buccal samples from MZ twins (including 10 samples that were previously assessed on HM450 [16]) with the aim to examine the sources of individual differences in DNA methylation obtained with EPIC and to validate the EPIC array in comparison with the HM450 array for buccal DNA samples. To this end, we examined: (1) the cellular content of buccal swabs based on DNA methylation profiles [21]; (2) the correlation between replicate measures of samples on the EPIC array and the correlation between samples measured on the EPIC and HM450 array (based on the common CpGs); (3) the correlation between MZ twins for genome-wide DNA methylation levels and for individual CpGs assessed by the EPIC array, the effect of variation in cellular proportions on MZ twin correlations and differences, and the reproducibility of methylation differences between MZ twins across different EPIC arrays; and (4) the effect of mQTLs in *cis*.

## Results

### Variation in cellular content of buccal swabs

**Fig. 1:**
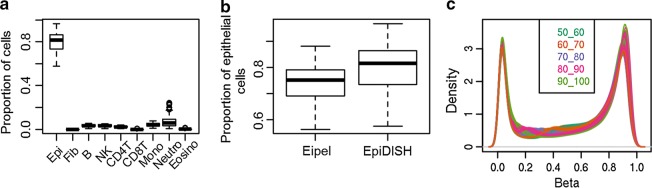
Cellular proportions of buccal swabs. **a** Estimated cellular proportions by HepiDISH. Epi = Epithelial cells, Fib = Fibroblasts, B = B cells, NK = natural killer cells, CD4T = CD4 + T cells, CD8T = CD8 + T cells, Mono = monocytes, Neutro = neutrophils, Eosino = eosinophils.** b** Epithelial cell proportions estimated by the method described by Eipel et al. and by EpiDISH. **c** Density plot of genome-wide methylation values of samples measured on EPIC, colored by epithelial cell % (categorized into 5 groups)

The cellular content of buccal swabs was estimated based on methylation profiles with Hierarchical Epigenetic Dissection of Intra-Sample-Heterogeneity (HepiDISH); a reference-based cell-type deconvolution algorithm [21]. Predicted epithelial cell percentages ranged from 57.6% to 96.7% (mean = 79.6%, Fig. 1a). Estimates for fibroblasts were zero for all samples as expected. Estimates of epithelial cell proportions derived by HEpiDISH correlated strongly with estimates derived by a different method by Eipel et al. [22] (*r* = 0.97, *p* value < 2.2 × 10^−16^). However, HEpiDISH seemed to provide additional discrimination in the higher range of epithelial cell percentages compared to the method by Eipel et al. (Fig. 1b) and has the advantage that it also allows to estimate proportions of leukocyte sub-types. HepiDISH estimates indicated that neutrophils were the most frequent leukocyte sub-type in buccal swabs (mean = 7.4%, range = 0.5–24.0%), followed by lymphocytes (B cells: mean = 3.2%, natural killer cells: mean = 3.4%, and CD4 + T cells: mean = 2.1%), and monocytes (mean = 4.0%). This pattern is comparable to findings from a microscopy-based method that counted broad classes of leukocytes in buccal swabs [23]. Estimates of CD8 + T cells and eosinophils were virtually zero.

Density plots illustrate that samples with different epithelial cell proportions show distinct genome-wide methylation profiles (Fig. 1c), and epithelial cell proportion correlated nearly perfectly (*r* = − 0.99) with principal component 1 (PC1) obtained by principal component analysis (PCA) on the genome-wide DNA methylation data. Epithelial cell proportions correlated moderately between MZ twins (*r* = 0.51 *p *= 1.8 × 10^−4^), which may reflect familial influences on adherence to the buccal swab collection protocol and familial influences on cells that are present in the mouth.

### Reproducibility of genome-wide methylation profiles on EPIC and comparison to HM450 array

**Fig. 2:**
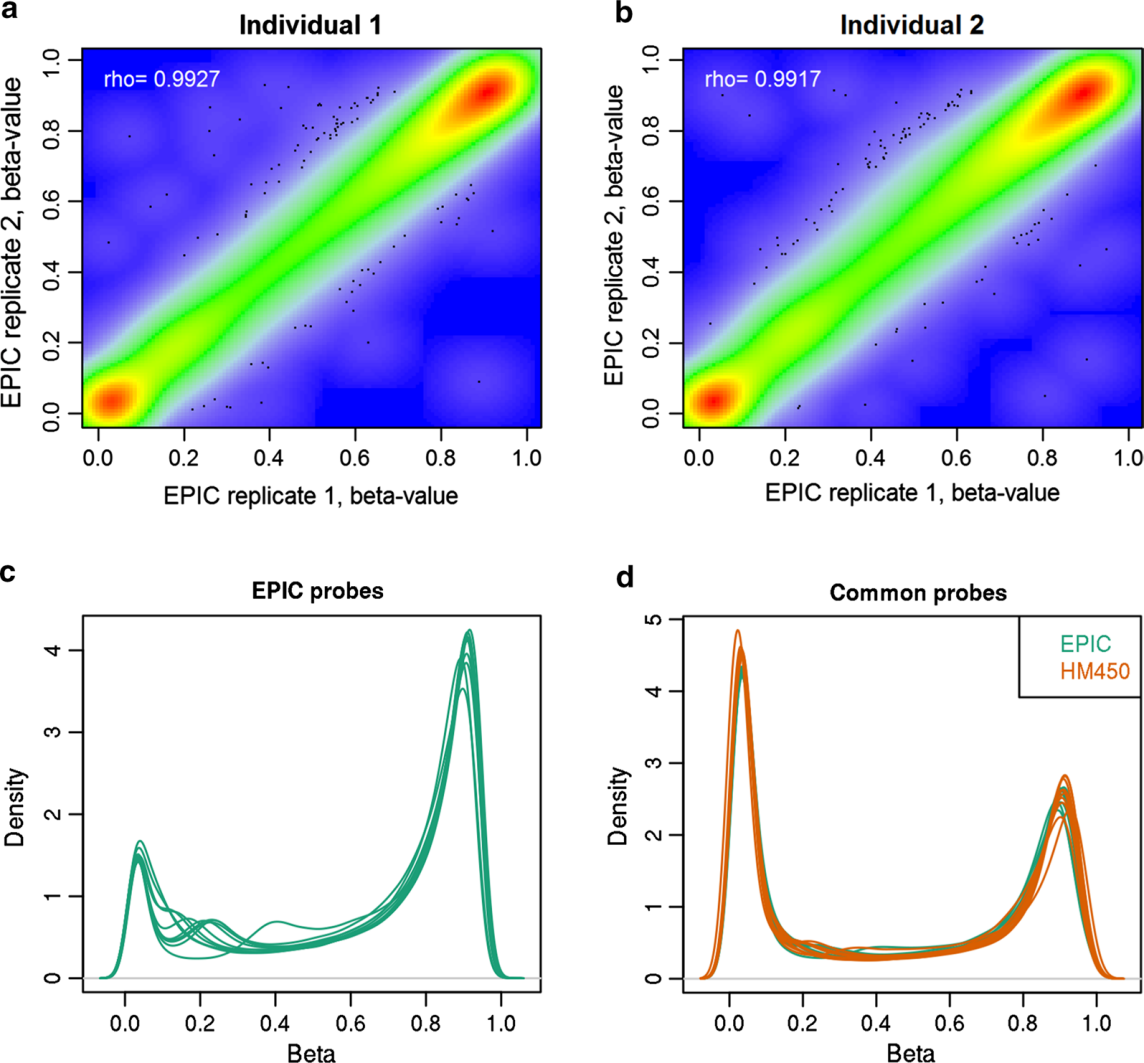
Methylation profiles of replicate samples on EPIC and matched samples on EPIC and HM450. **a**, **b** Scatterplots of methylation β-values of replicate samples from two individuals on EPIC. **c**, **d** Density plot of genome-wide DNA methylation values for ten buccal DNA samples that were assessed on EPIC and HM450.**c** Density plot of methylation probes that are unique to EPIC. **d** Density plot of methylation probes that are common to EPIC and HM450, for the same samples. Orange = HM450 arrays, green = EPIC arrays

For two individuals (MZ twins), a DNA sample was measured twice on EPIC using different BeadChip arrays to examine technical reproducibility of the EPIC array. Correlations between replicate samples on EPIC utilizing methylation β-values across 789,888 methylation sites were similar to previously published replicate correlations for DNA from other tissues (*r* = 0.9964 and *r* = 0.9976, Fig. 2a, b, Table 1) [3].

**Table 1:** Correlations between samples based on genome-wide DNA methylation profiles

Comparison	Pearson *r*			Spearman rho			Pearson *r*, standardized beta-values		
	Mean	Min	Max	Mean	Min	Max	Mean	Min	Max
Replicates on EPIC (2 pairs)	0.9970	0.9964	0.9976	0.9922	0.9917	0.9927	0.3972	0.3004	0.4941
Matched samples on EPIC and HM450 (10 pairs)	0.9942	0.9923	0.9954	0.9790	0.9768	0.9806	0.3064	0.0931	0.4372
MZ twins on EPIC (49 pairs)	0.9932	0.9797	0.9979	0.9884	0.9758	0.9938	0.3113	− 0.0786	0.7288
Unrelated pairs of samples on EPIC (1176 pairs)	0.9858	0.9451	0.9945	0.9810	0.9522	0.9902	− 0.0109	− 0.5118	0.4765

Next, we compared data obtained with the EPIC array to data obtained with the HM450 array for 10 DNA samples that were measured on both arrays. As previously observed in other tissues [3], the novel EPIC CpGs more often show intermediate methylation or hypermethylation (Fig. 2c) compared to CpGs that are common to EPIC and HM450 (Fig. 2d). We computed the correlation between methylation values of matched samples on EPIC and HM450 based on the overlapping CpGs. For all ten samples, DNA methylation profiles obtained by the different platforms correlated strongly (mean *r* = 0.9942, range = 0.9923–0.9954, Additional file 1: Figure S1), although not as strongly as two replicates on the EPIC array.

Importantly, correlations based on genome-wide methylation β-values were also large for pairs of DNA samples from unrelated subjects (mean *r* = 0.9858, range = 0.9451–0.9945), which also has been reported previously. Therefore, we also computed Pearson correlations between the normalized β-values that were standardized (z-scores) prior to computing the correlation (Table 1). While the correlations between unstandardized β-values are greatly influenced by the many CpGs with β-values close to the extremes (0 or 1), correlations between standardized β-values are not affected by this and are better suited to obtain a measure of the correlation between genome-wide DNA methylation profiles. Comparing the correlations based on standardized methylation β-values, we found that correlations were strongest between replicate samples on EPIC (mean *r* = 0.3972), followed by matched samples on EPIC and HM450 (mean *r* = 0.3064) and correlations between MZ twins (mean *r* = 0.3113), and correlations between DNA samples from unrelated subjects on EPIC were lowest (mean *r* = − 0.0109).

### Genome-wide resemblance of MZ twins

**Fig. 3:**
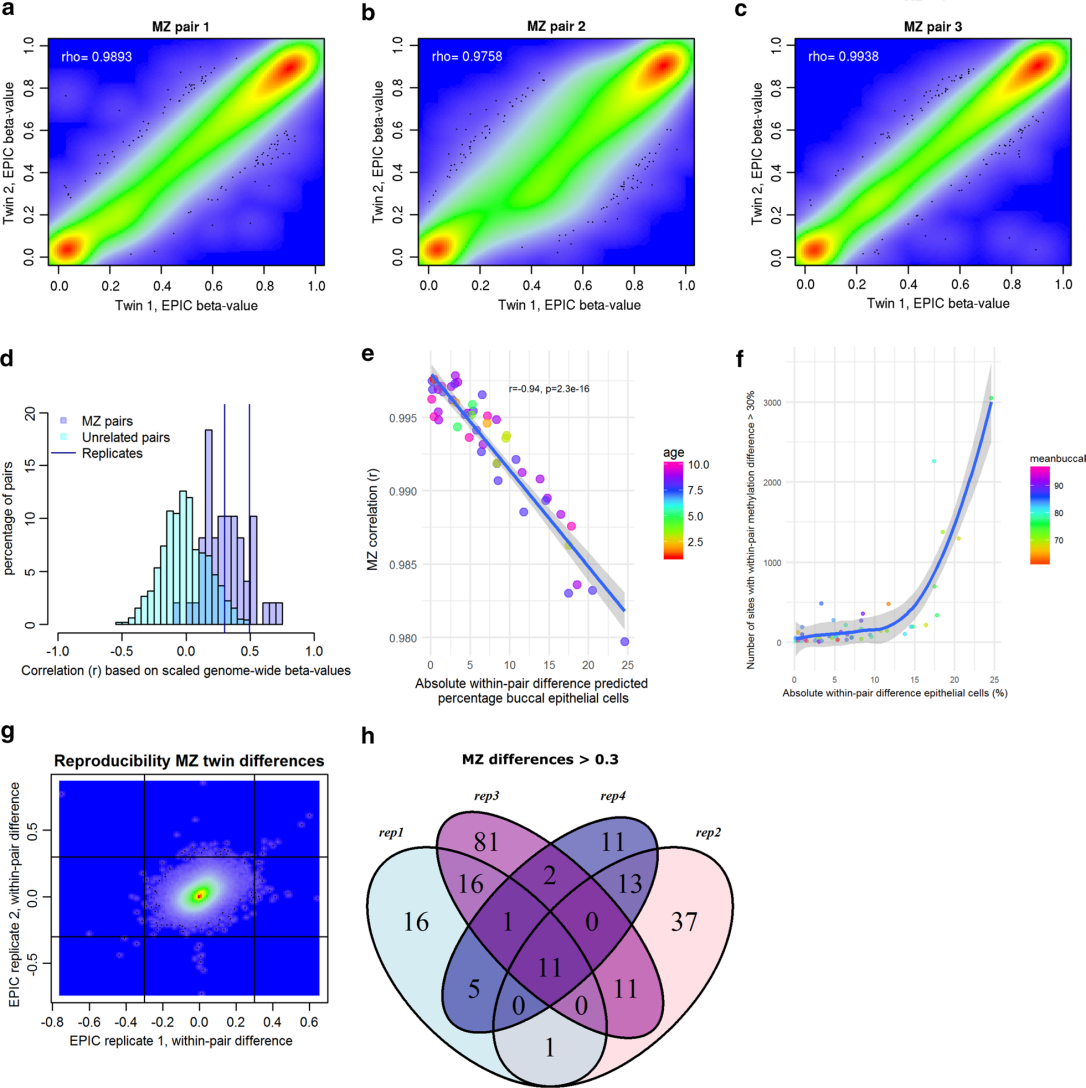
Genome-wide methylation profiles of MZ twin pairs based on buccal-derived DNA measured on the EPIC array. **a** Scatterplot showing genome-wide methylation β-values of co-twins of 1 exemplary twin pair (median correlation). **b** Scatterplot showing genome-wide methylation β-values of co-twins of 1 exemplary twin pair (lowest correlation). **c** Scatterplot showing genome-wide methylation β-values of co-twins of 1 exemplary twin pair (highest correlation). **d** Histogram of correlations between samples from: MZ twins (purple), unrelated subjects (green), and replicates (same DNA sample run twice on EPIC; blue lines). **e** The correlation between genome-wide methylation values of twins (r, *y*  axis) is plotted against the absolute within-pair difference in epithelial cell percentage % (*x* axis). Samples are colored by age at DNA collection. **f** Relationship between the number of CpGs with a methylation difference > 30% between MZ twins (*y* axis) and within-pair difference in proportion of epithelial cells(*x* axis). Colors denote the mean proportion of buccal cells of the two twin samples. **g** Within-pair differences in one MZ twin pair assessed twice on EPIC (technical replicates). The scatterplot shows two replicate measures of the within-pair difference of this twin pair. Horizontal and vertical lines indicate within-pair difference greater than 30%. **h** Venn diagram of the overlap across four replicate measures (rep1–rep 4) of within-pair methylation differences > 0.3 detected in one MZ twin pair assessed twice on EPIC array

Correlations for MZ pairs between genome-wide methylation β-values of twins obtained with the EPIC array were similar to previously published correlations based on HM450 (mean *r* = 0.9932, Fig. 3a–c) [16]. Correlations for MZ pairs based on standardized methylation values (mean *r* = 0.3113) tended to be lower than correlations between replicates (same DNA sample ran twice on EPIC), although for 14 pairs (29%), genome-wide methylation profiles of co-twins correlated as strongly as replicate measures of the same DNA (based on the comparison of the MZ twin correlation to the mean correlation of the two replicate pairs, Fig. 3d, Table 1). Absolute within-pair differences in epithelial cell proportion of MZ twins showed a strong negative correlation with MZ twin correlations of genome-wide methylation values (*r* = − 0.94, *p* = 2.2 × 10^−16^, Fig. 3e), indicating that differences in cellular content of buccal swabs are a major driver of differences between MZ twins in methylation profiles.

### Frequency and reproducibility of methylation differences within MZ pairs

For each MZ pair, we computed the within-pair difference in methylation β-values and counted the number of large differences (CpGs with a methylation difference larger than 30%). On average, 286 of such differences were observed per twin pair (median = 93, range 7–3051). The number of large methylation differences correlated, as expected, with discordance for cellular proportions (Fig. 3f). However, there were also twin pairs with very similar buccal epithelial cell proportions that still showed hundreds of large methylation differences (Fig. 3f).

For the twins who were measured twice on EPIC, we have four replicate measures of their within-pair methylation difference (Fig. 3g), which indicated 43–122 large differences, involving 205 CpGs in total. Of these CpGs, 11 were consistently detected at the threshold of 30% by all four measures of the within-pair difference (Fig. 3h). The correlation between replicate measures of within-pair differences across these 205 CpGs ranged from *r* = 0.19 to *r* = 0.66 for the four comparisons (mean *r* = 0.46).

### MZ twin correlation for individual CpGs

**Fig. 4:**
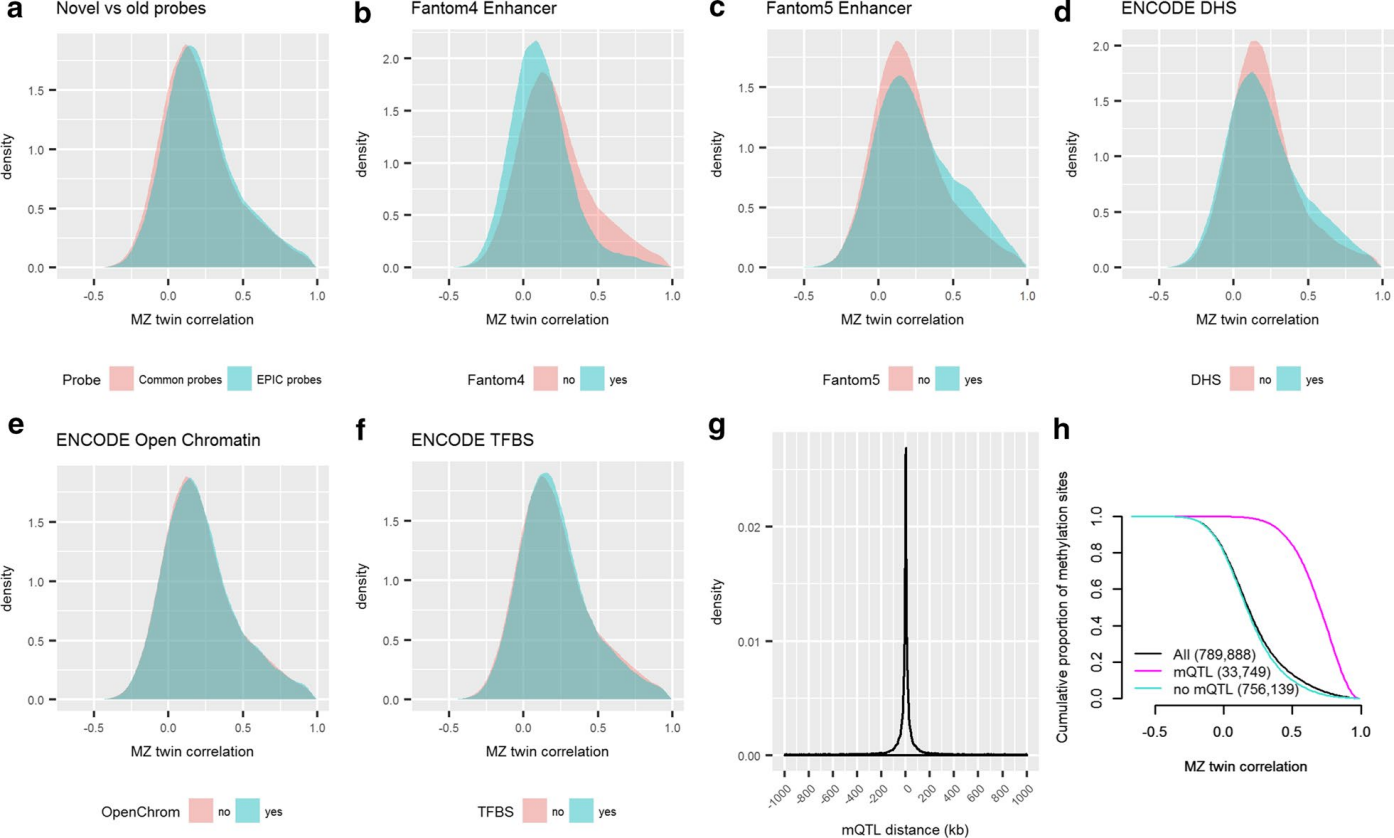
Distribution of MZ twin correlations for individual methylation sites assessed by the EPIC array as a function of probe category, functional elements, and mQTLs. **a** Distribution of MZ twin correlations for methylation probes that are common to EPIC and HM450 (pink) and for methylation probes that are unique to the EPIC array (blue). **b**–**f** Distribution of MZ twin correlations for methylation probes that overlap with (blue) or do not overlap with (pink) FANTOM4 enhancers (**b**), FANTOM 5 enhancers (**c**), Encode DHS (**d**), Encode open chromatin (**e**), Encode transcription factor binding sites (**f**). **g** Density of distances between methylation sites and the most strongly associated significant meQTL SNP. **h** MZ twin correlations as a function of mQTLs. The cumulative proportion of methylation sites (*y*-axis) at each MZ twin correlation (*x*-axis), for all genome-wide methylation sites (black), sites that are not significantly associated with an mQTL (blue) and sites that are associated with at least one significant mQTL (purple)

Next, we computed the correlation between MZ twins for individual CpGs after adjusting for cellular proportions that showed variation between samples: epithelial cells, neutrophils, B cells, natural killer cells, CD4 + T cells, and monocytes. The mean MZ twin correlation was 0.21 across all 789,888 autosomal EPIC CpGs after QC (Table 2). Correlations were slightly larger (Mann–Whitney *p* < 2.2 × 10^−16^) for the new EPIC probes (*N* = 383,066, mean *r* = 0.22) compared to the probes that are common to HM450 k and EPIC (*N* = 406,822, mean *r* = 0.20, Fig. 4a). Correlations obtained without adjustment for cellular proportions are presented in Additional file 1: Table S1. Based on visual inspection of the distribution of MZ twin correlations for CpGs located in various regulatory elements reported by the Encyclopedia of DNA Elements (ENCODE [24]) and Functional ANnoTation Of the Mammalian genome (FANTOM [25]) projects (Fig. 4b–f), MZ twin correlations tended to be larger for CpGs in FANTOM5 enhancers and ENCODE DNase I hypersensitive sites (DHSs), which were deliberately enriched among the novel probe content of the EPIC array. We tested for enrichment of cell-type-specific regulatory elements among CpGs with MZ twin correlations larger than 0.5 before adjusting for cell proportions (160,006 CpGs; 20.3%) and after adjusting for cell proportions (*N* = 104,845 CpGs; 13.3%), against a background of CpGs from the EPIC array with similar properties [26]. For both sets, based on ENCODE DHS data, we observed strongest enrichment of DHSs in epithelium cells (Additional file 2: Figure S2 and Additional file 3: Figure S3). This is expected because epithelial cells are the major cell type present in buccal swabs and confirm the quality of the data. Testing for overlap with 15 chromatin states from the Roadmap Epigenomics project revealed strongest enrichment in enhancers of epithelial tissues, with esophagus showing the strongest enrichment (Additional file 4: Figure S4 and Additional file 5: Figure S5). Of note, buccal epithelial cells are not included in either ENCODE or Roadmap, and of the available reference tissues, esophagus is the closest to buccal. Finally, testing for overlap with five core histone marks pointed at H3K4me3 in epithelial cell types and tissues as the top enriched histone mark (Additional file 6: Figure S6, Additional file 7: Figure S7). H3Kme3 is associated with transcriptional start sites of actively transcribed genes. Without adjustment for cellular proportions, we also observed a weak signal of enrichment of leukocyte elements (and a number of other tissues; Additional file 2: Figure S2, Additional file 4: Figure S4, Additional file 6: Figure S6). After adjusting for cellular proportions, epithelium was still the most strongly enriched, while the signal for leukocytes and other tissues was generally reduced (Additional file 3: Figure S3, Additional file 5: Figure S5, Additional file 7: Figure S7).

**Table 2:** MZ twin correlations for DNA methylation level at all autosomal methylation sites assessed by the EPIC array

Probes	Min	Median	Mean	Max
All EPIC probes (789888)	− 0.67	0.18	0.21	0.99
Novel EPIC probes (383066)	− 0.58	0.19	0.22	0.99
Common probes (406822)	− 0.67	0.16	0.20	0.99

Results after adjusting for cellular composition are displayed

### mQTL analysis

We performed *cis* mQTL analysis to identify genetic variants associated with methylation levels at sites interrogated by the EPIC array using genome-wide imputed SNP data (1000 Genomes; 1000G) from 84 twins. This analysis identified 1,296,323 significant associations (FDR 5%), involving 33,749 methylation sites and 616,029 genetic variants. Methylation sites were associated with 1–3375 genetic variants (mean = 38, median = 13), and genetic variants were associated with 1–45 methylation sites (mean = 2, median = 1). As observed in previous mQTL studies, the distance between the genetic variant and methylation site was typically small (median distance = 18 kb between methylation site and the strongest associated SNP; Fig. 4g). Importantly, 52.9% of the detected methylation sites affected by mQTLs (*N* = 17,852) are novel EPIC probes that were not previously interrogated by the HM450 array. Of the genetic variants associated with novel EPIC probes, 293,047 (65.9%) were not significantly associated with any of the probes covered by HM450, illustrating the power of EPIC to reveal novel mQTL targets. Significant mQTLs affected 4.7% of EPIC probes and 3.9% of HM450 probes, which represents a significant enrichment of mQTL associations for novel EPIC probes (*x*^2^ = 273.3, *df* = 1, *p* value < 2.2 × 10^−16^). Of the 33,749 methylation sites with a significant mQTL in our study of buccal samples, 15,897 were also interrogated by the HM450 array, and 7356 of these sites (46.3%) were previously identified as being associated with genetic variants in blood by a large mQTL study (*N* = 3841 samples) by the Biobank-based Integrative Omics Study consortium that applied the HM450 array [17].

Methylation sites affected by one or more mQLs were characterized by substantially larger MZ twin correlations (mean *r* = 0.68, SD = 0.17) compared to methylation sites without significant mQLs (mean *r* = 0.19, *SD* = 0.23), Mann–Whitney *p* < 2.2 × 10^−16^ (Fig. 4h). Finally, we tested for enrichment of cell-type-specific regulatory elements among methylation sites with significant mQTLs, including DHS from ENCODE, and 5 core histone marks, and 15 chromatin states from the Epigenomics Roadmap Project. The analysis of DHS revealed the strongest enrichment of epithelial cell DHSs (Additional file 8: Figure S8), chromatin states pointed at epithelial cell enhancers as the top enriched category (Additional file 9: Figure S9), and the top enriched histone mark was H3K4me3 in keratinocytes (Additional file 10: Figure S10), highly similar to the pattern displayed by methylation sites with large MZ twin correlations.

## Discussion

We assessed DNA methylation in buccal swabs from monozygotic twins to examine the sources of individual differences in DNA methylation for sites interrogated by the EPIC array and to validate the EPIC array in comparison with the HM450 array for buccal DNA samples. Correlations between replicates on EPIC for genome-wide methylation profiles were similar to previously published correlations for DNA from other tissues [3]. The same was true for correlations between DNA samples measured on EPIC and HM450, based on genome-wide common methylation sites present on both arrays. For individual methylation sites, the genome-wide average MZ twin correlation of DNA methylation level obtained with the EPIC array was 0.21, and MZ twin correlations were slightly larger for the novel EPIC probes (mean *r* = 0.22) compared to the probes that are common to EPIC and HM450 (mean *r* = 0.20). In line with this pattern, we observed a small enrichment of mQTL effects among methylation sites interrogated by novel EPIC probes. The novel EPIC probe content was designed to cover potential enhancers identified in a variety of tissues and cell types by FANTOM5 [25] and ENCODE [27]. When we analyzed the distribution of methylation sites with a large correlation between MZ twins (*r* > 0.5) across cell-type specific regulatory elements, we found the strongest enrichment in epithelial cell enhancers, DHS and H3K4me3; the histone mark associated with transcriptional start sites of actively transcribed genes. The same was true for methylation sites that showed the strongest mQTL effects. These findings illustrate that correlations between MZ twins for DNA methylation level and mQTL effects are stronger for sites located in regulatory elements connected to active regions of the major cell type from which DNA was extracted. These sites are better covered by novel EPIC probes.

To allow for better comparison of the correlations between genome-wide DNA methylation profiles of replicate samples, samples from MZ twins, and unrelated samples, we obtained correlations based on standardized methylation beta-values. This method illustrated the striking similarity of MZ twins for genome-wide methylation profiles in comparison with genome-wide methylation profiles from unrelated pairs of individuals. Notably, 29% of MZ pairs correlated as strongly as technical replicate measures of the same DNA. This implies that the genome-wide methylation differences between some pairs may not exceed the amount of variation that can result from (unsystematic) technical noise, which was not entirely unexpected. Of note, this does not rule out that true methylation differences may be present in such pairs. By comparing large within-pair methylation differences obtained by replicate measures of one MZ twin pair, we found that some large methylation differences were consistently detected by multiple EPIC BeadChip Arrays.

We, and others, have previously used the HM450 array to assess genome-wide DNA methylation in buccal swabs from twins [15, 16]. In our previous study, we reported a mean correlation between MZ twins of 0.31 across all HM450 probes [16]. This estimate lies within the range of correlations that we obtained with the EPIC array in the current study when we did not correct for cellular proportions (mean *r* = 0.30 across all probes, mean *r* = 0.32 for novel EPIC probes, and mean *r* = 0.28 for common probes). Previously, we did not correct for cellular proportions with HEpiDISH since this method was not available at the time. The reduction of the mean MZ twin correlation after cell type correction implies that part of the variation in DNA methylation profiles that is shared by MZ twins is due to resemblance of MZ twins with respect to cellular proportions. We found that the correlation between epithelial cell proportions of buccal samples from MZ twins was 0.51. This correlation may reflect familial influences on adherence to the buccal swab collection protocol and familial influences on cells that are present in the mouth. Familial influences include genetic and shared environmental influences.

Buccal swabs offer potential advantages to human epigenetic studies. Firstly, they contain epithelial cells, which are ectodermal, and may therefore be a better surrogate tissue for ectodermal tissues such as the brain compared to other peripheral tissues such as blood [28, 29]. Secondly, buccal swab collection is noninvasive, making it convenient for large-scale human epigenetic studies, especially in children. Buccal swabs are a relatively homogeneous tissue, in the sense that they contain only two major cell types (buccal epithelial cells and leukocytes). However, based on a previously published method to predict cellular proportions in samples such as buccal swabs [21], we observed fairly large variation in the relative proportions of epithelial cells and leukocytes between samples (range of predicted buccal cell percentage between samples: 57.6% to 96.7%). Similar variation in the proportion of epithelial cells in buccal swab samples was also recently noted by two other studies: one study that utilized a microscopy-based method [23] to obtain cell counts and one study that described the cell-type deconvolution method that we also applied in the current study [21]. Differences in cellular composition were the most important contributor to variation in genome-wide DNA methylation profiles across samples, including differences between MZ twins. These findings highlight the importance to adjust for cellular composition in DNA methylation studies of buccal swabs, as is commonly recognized in studies of more frequently studied tissues in epigenetic studies, such as whole blood. After adjusting for predicted cellular proportions, the genome-wide average MZ twin correlation was reduced, and we observed negligible enrichment of leukocyte regulatory elements among sites with a larger MZ twin correlation. This confirms the effectiveness of this correction and indicates that large MZ twin correlations for methylation sites are (primarily) driven by similarity for DNA methylation levels that vary within buccal epithelial cells.

It is possible that the relative proportion of buccal epithelial cells that is harvested might be affected by how well individuals adhere to the instructions of our protocol and that different protocols or tools (e.g., cotton swabs versus flocked swabs) may yield different proportions of buccal epithelial cells. The epithelial cell proportions observed in our study (mean = 79.6%) are intermediate of reports from three previous studies [21–23]. Our estimates are slightly lower than the estimates reported by Theda et al. (mean = 90% in children and 83% in adults) [23], who used a microscopy-based method to quantify cellular proportions, and they are higher than the estimates reported by Eipel et al. (mean = 65%; based on microscopy [22]) and Zheng et al. (mean ~ 50% according to Fig. 5) [21]. The estimates by Zheng et al. are based on the same reference-based cell type deconvolution method as applied by us. Of note, currently available cell-type deconvolution algorithms do not allow to distinguish between different sub-types of epithelial cells present in buccal swabs. The epithelial cells present in buccal swabs may be classified into three sub-types, namely intermediate squamous cells, non-keratinous superficial squamous cells (derived from the surface layer of the inner cheek), and keratinous superficial squamous cells (derived from the surface layer of the gingiva) [23]. Novel methods that would allow to estimate these sub-types may be valuable.

This study has several strengths and limitations. This is the first study that has measured DNA methylation with the EPIC array on DNA obtained from buccal swabs, the first study that has measured DNA methylation with the EPIC array in MZ twins, and the first to our knowledge to perform mQTL analysis on buccal samples. Correlations between MZ twins provide an indication of the relative importance of familial factors (genetic variation and shared environment combined) versus the importance of environmental and stochastic influences to inter-individual variation in methylation levels. Future studies that also include DZ twins will allow to estimate the heritability of DNA methylation levels for EPIC probes and to estimate the variance due to common environment. It also remains to be investigated whether other tissues show a similar pattern of higher MZ twin correlations for DNA methylation at novel EPIC probes and at tissue-specific regulatory elements.

## Conclusions

We conclude that the performance of EPIC and HM450 arrays on buccal-derived DNA is similar and that the total contribution of familial factors (DNA sequence and shared environment) to individual differences in DNA methylation and the effect of mQTLs is larger for novel EPIC probes, especially for probes located in regulatory elements connected to active regions specific to the main cell type of the investigated tissue. Our findings highlight the value of the novel EPIC probe content for interrogating biologically meaningful differences in DNA methylation level between samples and for detecting novel mQTL targets that are not covered by HM450 probes. The results of this study provide a first resource of genetic effects on DNA methylation for the EPIC array in buccal tissue from children.

## Methods

### Subjects and samples

The subjects take part in longitudinal studies from the Netherlands Twin Register (NTR) [30]. For the current study, we selected 107 buccal samples from 105 monozygotic twins (52 complete pairs and 1 incomplete pair, 58% males, mean age at DNA collection = 7 years, range = 1–10). For two twins (one pair), a technical replicate measure on EPIC was obtained by running the same DNA twice on the EPIC array (on different BeadChip Arrays). For 10 twins, methylation data had been generated before with the HM450 array on the same DNA sample [16]. Genome-wide SNP data from genotype arrays were available for 90 twins. Four twins were identified as ethnic outliers based on genome-wide SNP data and excluded from the mQTL analysis, resulting in a total sample size of 86 twins in the mQTL analysis. This study is embedded in a larger project on childhood aggression and consists of a selected group of twins who score high or low on aggression. Participants could indicate if they wished to be informed of the results of zygosity testing. Zygosity testing, based on a set of SNPs and VNTRs, as described previously [30], confirmed that all pairs were MZ.

### Buccal DNA collection for DNA methylation assays

The procedures of buccal swab collection [31] have been described previously. In short, 16 cotton mouth swabs were individually rubbed against the inside of the cheek by the participants and placed in four separate 15-mL conical tubes (four swabs in each tube) containing 0.5 mL STE buffer (100 mM sodium chloride, 10 mM Tris hydrochloride (pH 8.0) and 10 mM ethylenediaminetetraacetic acid) with proteinase K (0.1 mg/mL) and sodium dodecyl sulfate (SDS) (0.5%) per swab. Individuals were asked to refrain from eating or drinking 1 h prior to sampling. High molecular weight genomic DNA was extracted from the swabs using standard DNA extraction techniques. The DNA samples were quantified using the Quant-iT PicoGreen dsDNA Assay Kit (ThermoFisher Scientific, Waltham, MA, USA).

### Infinium MethylationEPIC BeadChip data

DNA methylation was assessed with the Infinium MethylationEPIC BeadChip Kit (Illumina, San Diego, CA, USA) [2]. A total of 500 ng of genomic DNA from buccal swabs were bisulfite-treated using the ZymoResearch EZ DNA Methylation kit (Zymo Research Corp, Irvine, CA, USA). The Infinium HD Methylation Assay (amplification, fragmentation, precipitation, hybridization, wash, extension, staining, and imaging) was performed at the Avera Institute for Human Genetics according to the manufacturer’s explicit specifications.

### DNA methylation quality control

#### Overview

Quality control (QC) and normalization of the methylation data were performed using a pipeline developed by the Biobank-based Integrative Omics Study (BIOS) consortium [32], which includes sample quality control using the R package MethylAid [33] and probe filtering and functional normalization as implemented in the R package DNAmArray. MethylAid was applied with the default array-specific quality filter thresholds for EPIC and HM450 arrays. The identity of replicate samples on EPIC, samples that were measured on EPIC and HM450, and the zygosity of twins was verified with the R package omicsPrint [34].

#### EPIC arrays

First, the EPIC array data were processed separately. Out of all EPIC arrays, five DNA samples (4.8%) produced sub-optimal sample level QC. MethylAid quality control plots are provided in Additional file 1: Figure S11–S15. Functional normalization was performed based on five control probe PCs. A screeplot of control probe PCs is shown in Additional file 1: Figure S16a. The following probe filters were applied: Probes were set to missing (NA) in a sample if they had an intensity value of exactly zero, detection *p* value > 0.01, or bead count < 3. Probes were excluded from all samples if they mapped to multiple locations in the genome, if they overlapped with a single nucleotide polymorphism (SNP) or Insertion/Deletion (INDEL), or if they had a success rate < 0.95 across samples. Annotations of ambiguous mapping probes (based on an overlap of at least 47 bases per probe) and probes where genetic variants (SNPs or INDELS) with a minor allele frequency > 0.01 in Europeans overlap with the targeted CpG or single base extension site (SBE) were obtained from Pidsley et al. [3]. After probe filtering, the success rate of probes for each sample was checked: All samples had a success rate above 0.95. Only autosomal methylation sites were analyzed, leaving 789,888 out of 865,859 sites for analysis, including 406,822 CpGs that are also interrogated by the HM450 array and 383,066 novel CpGs. PCA was performed with DNAmArray prior to and after normalization, and the correlation of the first ten PCs with technical and biological variables (e.g., age, sex, epithelial cell proportion) was computed to check for batch effects and biological correlates of variation in genome-wide methylation patterns. These analyses indicated that normalization successfully reduced variation related to technical factors such as 96-well plate position and the location of the sample on the EPIC array, and that biological factors (cellular composition of samples and sex) are the most important drivers of variation in genome-wide methylation levels (as illustrated by their strong correlation with PC1 and PC2, Additional file 1: Figure S17 and Figure S18). OmicsPrint confirmed the identity of samples on EPIC, samples that were measured on EPIC and HM450, and the zygosity of twins (Additional file 1: Figure S19 and Figure S20).

#### Combined dataset EPIC and HM450 arrays

In a second step, all EPIC arrays and HM450 arrays were processed and normalized jointly based on the common probe content of the EPIC and 450 k array. We first applied sample QC in MethylAid, separately, on the HM450 and EPIC arrays. Next, the raw signal intensity data (RGsets) from the EPIC and HM450 arrays were merged with the minfi package function combineArrays() to create a virtual HM450 array [35]. After merging, we applied the same filtering and normalization steps as described above. Functional normalization was performed based on 5 control probe PCs. A screeplot of control probe PCs is shown in Additional file 1: Figure S16b. This dataset was used for the comparison of the 10 matched samples that were measured on EPIC and HM450 and included 407,395 methylation sites after QC.

### Cellular proportions

Cellular proportions were predicted with Hierarchical Epigenetic Dissection of Intra-Sample-Heterogeneity (HepiDISH) with the RPC method (reduced partial correlation), as described by Zheng et al. [21] and implemented in the R package EpiDISH. HepiDISH is a cell-type deconvolution algorithm that was specifically developed for estimating cellular proportions in epithelial tissues based on genome-wide methylation profiles and makes use of reference DNA methylation data from epithelial cells, fibroblast and seven leukocyte sub-types. We also used the method described by Eipel et al. [22] to predict epithelial cell proportions based on two CpGs (cg07380416 and cg20837735). It was previously reported that estimates obtained by this method correlated strongly with buccal epithelial cell counts based on hematoxylin/eosin staining (*r*^2^ = 0.94) [22]. Both methods were applied to the data after data QC.

### Methylation data annotation

The following genomic annotations were obtained from the EPIC manifest file provided by Illumina (MethylationEPIC_v-1-0_B4.csv): locations of CpG islands, ENCODE DNase I hypersensitive sites (DHSs), ENCODE transcription factor binding sites (TFBSs), open chromatin, FANTOM4 enhancers and FANTOM5 enhancers.

### Genome-wide SNP data

Genotyping was carried out on several genome‐wide SNP micro‐arrays [36]. SNP genotype pre-imputation quality control, haplotype phasing, and 1000 Genomes imputation have been described previously by Lin et al. [36].

### Analyses

#### Correlations between samples

To examine the similarity of genome-wide DNA methylation profiles between pairs of observations (technical replicates on EPIC, matched samples on EPIC and HM450, samples from MZ twins, and samples from unrelated pairs of individuals), we computed the correlations between normalized β-values. We present three different types of correlations to allow for comparison with previously published correlations. Firstly, we computed Pearson correlations (r) and Spearman correlations (rho) between normalized β-values (across all CpGs, i.e., CpGs are cases), as reported in previous studies. We also computed Pearson correlations between the normalized β-values that were standardized (z-scores) prior to computing the correlation. While the correlations between unstandardized β-values are greatly influenced by the many CpGs with β-values close to the extremes (0 or 1), correlations between standardized β-values are not affected by this and are better suited to obtain a measure of the correlation between genome-wide DNA methylation profiles.

#### MZ twin correlations for individual CpGs

Secondly, for each CpG, the Pearson correlation (r) was computed between the β-value of Twin 1 and the β-value of Twin 2 (across all MZ twin pairs, i.e., MZ twin pairs are cases), as a measure of the similarity of the methylation level of a CpG in MZ twins. These correlations were computed on the normalized methylation β-values and on the residuals derived after adjusting for covariates. Mann–Whitney tests were performed to test for differences in the MZ twin correlation between novel EPIC probes that are common to EPIC and HM450 and between probes with significant mQTLs and without significant mQTLs, with the R wilcox.test() function.

#### Adjustment for covariates

DNA methylation β-values were adjusted for covariates by running linear models with the R function lm. Residuals were saved and used as input for computing correlations between MZ twins for individual CpGs and for the mQTL analysis. Prior to calculating the MZ twin correlations for individual CpGs, methylation data were adjusted for cellular proportions of buccal swabs estimated by HepiDISH to account for variation in cellular composition between samples from different twins. We adjusted for the following cellular proportions that showed variation between samples: epithelial cells, neutrophils, B cells, natural killer cells, CD4 + T cells, and monocytes. Prior to the mQTL analysis, methylation data were adjusted for the same cellular proportions plus sex, age, and the first ten principal components (PCs) obtained from genome-wide SNP data to account for population structure within the Netherlands.

#### Within-pair differences MZ twins

For each twin pair, the within-pair difference in DNA methylation β-value (Δmethylation) was computed for each CpG. Next, the number of CpGs with a large within-pair difference per twin pair was counted, which we defined as a difference larger than 0.3 (i.e., a difference in methylation percentage larger than 30%). Data from the pair of twins who were measured twice on EPIC were used to examine the reproducibility of large within-pair methylation differences. Specifically, we counted the overlap of CpGs with large methylation differences detected by replicate measures and computed the correlation between Δmethylation obtained by replicate measures.

#### *Cis* mQTL analysis

EPIC methylation data and genome-wide SNPs (1000G imputation) from 86 MZ twins were used for *cis* methylation (m)QTL analysis. In this analysis, all associations between genetic variants and methylation sites within a distance < 1 M base pairs (Mb) were computed. After adjusting for covariates, residual data for each methylation site were quantile-normalized prior to mQTL analysis. Imputed SNP genotypes were coded into reference allele dosage format and filtered at MAF > 0.01, HW *P *> 1E − 04, MAC > 10, and imputation *r*^2^ > 0.8, resulting in 2,846,659 remaining SNPs for mQTL analysis. *Cis* mQTL effects were detected with a linear model approach using MatrixeQTL [37] with methylation level as dependent variable and SNP genotype values as independent variable. To account for relatedness of the MZ twins, 10 permutations were performed wherein each permutation the relatedness was preserved (i.e., in each permutation the genotypes of the MZ twin pairs were assigned the methylation values of a random MZ twin pair), using the permutation approach previously applied in Jansen et al. [38] and Bonder et al. [17]: for each permutation, the complete *cis* mQTL analysis was repeated. The *P* value threshold for rejecting at FDR < 0.05 was computed based on these permutations: by identifying the P value threshold for which the total number of methylation sites with a significant mQTL in the permuted data divided by the total number of methylation sites with a significant mQTL in the unpermuted data was 0.05. Similar to what was observed in Fehrman et al. [39], only 10 permutations were needed to have the *P* value threshold corresponding to FDR < 5% converging. The P value threshold corresponding to FDR < 5% was 5.5 × 10^−6^. Of note, the mQTL P values computed in the mQTL analysis are based on the complete sample with related subject and thus are too liberal; however, the FDR takes into account the family structure and should be used to draw conclusions. The reported betas from the linear models can be correctly estimated from samples containing related subjects.

### Overlap with previous mQTL findings

Methylation sites that were previously reported to be associated with *cis* mQTLs in blood were obtained from the BIOS consortium [17]. This mQTL study analyzed HM450 array data from 3841 whole blood samples.

#### Enrichment of cell-type-specific regulatory elements

We tested if methylation sites with a large correlation in MZ twins (r > 0.5) and methylation sites strongly affected by (an) mQTL(s) were enriched within cell-type-specific regulatory elements (consolidated Roadmap Epigenomics data on histone marks and chromatin states [40], and DHSs from the ENCODE project [24]) with eFORGE [26]. This analysis can provide insight into cell-type-specific signals and into confounding by variation in cellular proportions between samples. If MZ twin correlations are confounded by cellular proportions, meaning that methylation sites with a large correlation are those sites that are differentially methylated between the major cell types present in buccal swabs (buccal epithelial cells and leukocytes), we expect to see enrichment of both epithelial and leukocyte-specific regulatory elements. As input list for eFORGE, we first randomly selected 1000 methylation sites from the total set of methylation sites with a correlation > 0.5 between MZ twins without adjusting for buccal epithelial cell proportion. Next, to verify the effectiveness of adjusting for buccal epithelial cell proportion, we ran eFORGE on an input list of methylation sites with a correlation > 0.5 after adjusting for cellular proportions (again randomly selecting 1000 CpGs from the total set). Third, we ran eFORGE on methylation sites with the strongest mQTL(s), by selecting the top 1000 methylation sites with the lowest mQTL P value. The analysis of histone marks tested for enrichment of five core marks [40]: histone H3 lysine 27 trimethylation (H3K27me3), associated with polycomb repression, H3 lysine 4 monomethylation (H3K4me1), associated with enhancer regions, H3 lysine 4 trimethylation (H3K4me3), associated with promoter regions, H3 lysine 36 trimethylation (H3K36me3), associated with transcribed regions, and H3 lysine 9 trimethylation (H3K9me3), associated with heterochromatin regions. The analysis of chromatin states tested for enrichment of 15 chromatin states (8 active states and 7 repressed states) [40], including: active transcriptional start site (TSS), flanking active TSS, transcribed at a gene’s 5′ and 3′ end, strong transcription, weak transcription, genic enhancers, enhancers, ZNF genes & repeats, heterochromatin, bivalent/poised TSS, flanking bivalent TSS/enhancer, bivalent enhancer, repressed polycomb, weak repressed polycomb, quiescent/low.

## Additional files

**Additional file 1.** **Table S1:** MZ twin correlations for DNA methylation level at all autosomal methylation sites, without adjustment for cellular composition. **Figure S1:** Scatterplots of methylation β-values of matched samples on EPIC and HM450. **Figure S11**: Quality control plot of bisulfite conversion. **Figure S12:** Quality control plot of overall sample quality based on sample-dependent control probes (Non-Polymorphic quality control probes). **Figure S13:** Quality control plot of the median Methylated versus Unmethylated signal intensity. **Figure S14:** Quality control plot based on sample-independent hybridization control probes. **Figure S15:** Quality control plot showing the proportion of probes with a detection p-value < 0.01 within samples. **Figure S16:** Scree plots of PCs based on control probes. **Figure S17:** Heatmap of the correlations of technical and biological variables with PCs based on the genome-wide methylation data prior to normalization. **Figure S18:** Heatmap of the correlations of technical and biological variables with PCs based on the genome-wide methylation data after functional normalization. **Figure S19:** IBS mean-variance plot from omicsPrint of samples measured on EPIC. **Figure S20:** IBS mean-variance plot from omicsPrint of matched samples measured on EPIC and HM450.

**Additional file 2.** DHS enrichment for methylation sites with large MZ twin correlation, unadjusted for cellular composition.

**Additional file 3.** DHS enrichment for methylation sites with large MZ twin correlation, adjusted for cellular composition.

**Additional file 4.** Chromatin state enrichment for methylation sites with large MZ twin correlation, unadjusted for cellular composition.

**Additional file 5.** Chromatin state enrichment for methylation sites with large MZ twin correlation, adjusted for cellular composition.

**Additional file 6.** Histone H3 mark enrichment for methylation sites with large MZ twin correlation, unadjusted for cellular composition.

**Additional file 7.** Histone H3 mark enrichment for methylation sites with large MZ twin correlation, adjusted for cellular composition.

**Additional file 8.** DHS enrichment for methylation sites with the strongest mQTLs.

**Additional file 9.** Chromatin state enrichment for methylation sites with the strongest mQTLs.

**Additional file 10.** Histone H3 mark enrichment for methylation sites with the strongest mQTLs.

## Abbreviations

mQTL: Methylation quantitative trait locus
MZ twin: Monozygotic twin
HM450 array: Illumina HumanMethylation450 BeadChip
EPIC array: MethylationEPIC BeadChip
WGBS: Whole-genome bisulfite sequencing
CRP: C-reactive protein
H3K27me3: Histone H3 lysine 27 trimethylation
H3K4me1: Histone H3 lysine 4 monomethylation
H3K4me3: Histone H3 lysine 4 trimethylation
H3K36me3: Histone H3 lysine 36 trimethylation
H3K9me3: Histone H3 lysine 9 trimethylation
PC: Principal component
PCA: Principal component analysis
HepiDISH: Hierarchical Epigenetic Dissection of Intra-Sample-Heterogeneity
ENCODE: Encyclopedia of DNA Elements
FANTOM: Functional ANnoTation Of the Mammalian genome
DHS: DNase I hypersensitive site
1000G: 1000 Genomes
SNP: Single nucleotide polymorphism
INDEL: Insertion/Deletion
TFBS: Transcription factor binding site
