# Supplementary material for: Genome-wide analysis of DNA methylation in buccal cells: a study of monozygotic twins and mQTLs
Source: Epigenetics Chromatin. 2018 Sep 25;11:54. doi: 10.1186/s13072-018-0225-x (PMC6156977; doi:10.1186/s13072-018-0225-x)
Supplement: Supplementary file 1 — Additional file 1. Table S1: MZ twin correlations for DNA methylation level at all autosomal methylation sites, without adjustment for cellular composition. Figure S1: Scatterplots of methylation β-values of matched samples on EPIC and HM450. Figure S11: Quality control plot of bisulfite conversion. Figure S12: Quality control plot of overall sample quality based on sample-dependent control probes (Non-Polymorphic quality control probes). Figure S13: Quality control plot of the median Methylated versus Unmethylated signal intensity. Figure S14: Quality control plot based on sample-independent hybridization control probes. Figure S15: Quality control plot showing the proportion of probes with a detection p-value < 0.01 within samples. Figure S16: Scree plots of PCs based on control probes. Figure S17: Heatmap of the correlations of technical and biological variables with PCs based on the genome-wide methylation data prior to normalization. Figure S18: Heatmap of the correlations of technical and biological variables with PCs based on the genome-wide methylation data after functional normalization. Figure S19: IBS mean-variance plot from omicsPrint of samples measured on EPIC. Figure S20: IBS mean-variance plot from omicsPrint of matched samples measured on EPIC and HM450. [file 13072_2018_225_MOESM1_ESM.pdf]

Supplemental Tables

Table S1 MZ twin correlations for DNA methylation level at all autosomal methylation sites, without adjustment for cellular composition

Probes	Min	Median	Mean	Max
All EPIC (789888)	-0.65	0.29	0.30	1.0
Novel EPIC probes (383066)	-0.55	0.32	0.32	1.0
Common probes (406822)	-0.65	0.27	0.28	1.0

Supplemental Figures

Figure S1 Scatterplots of methylation β-values of matched samples on EPIC and HM450. The x-axis displays the β-values obtained by the EPIC array and the y-axis displays the β-values obtained by the HM450 array

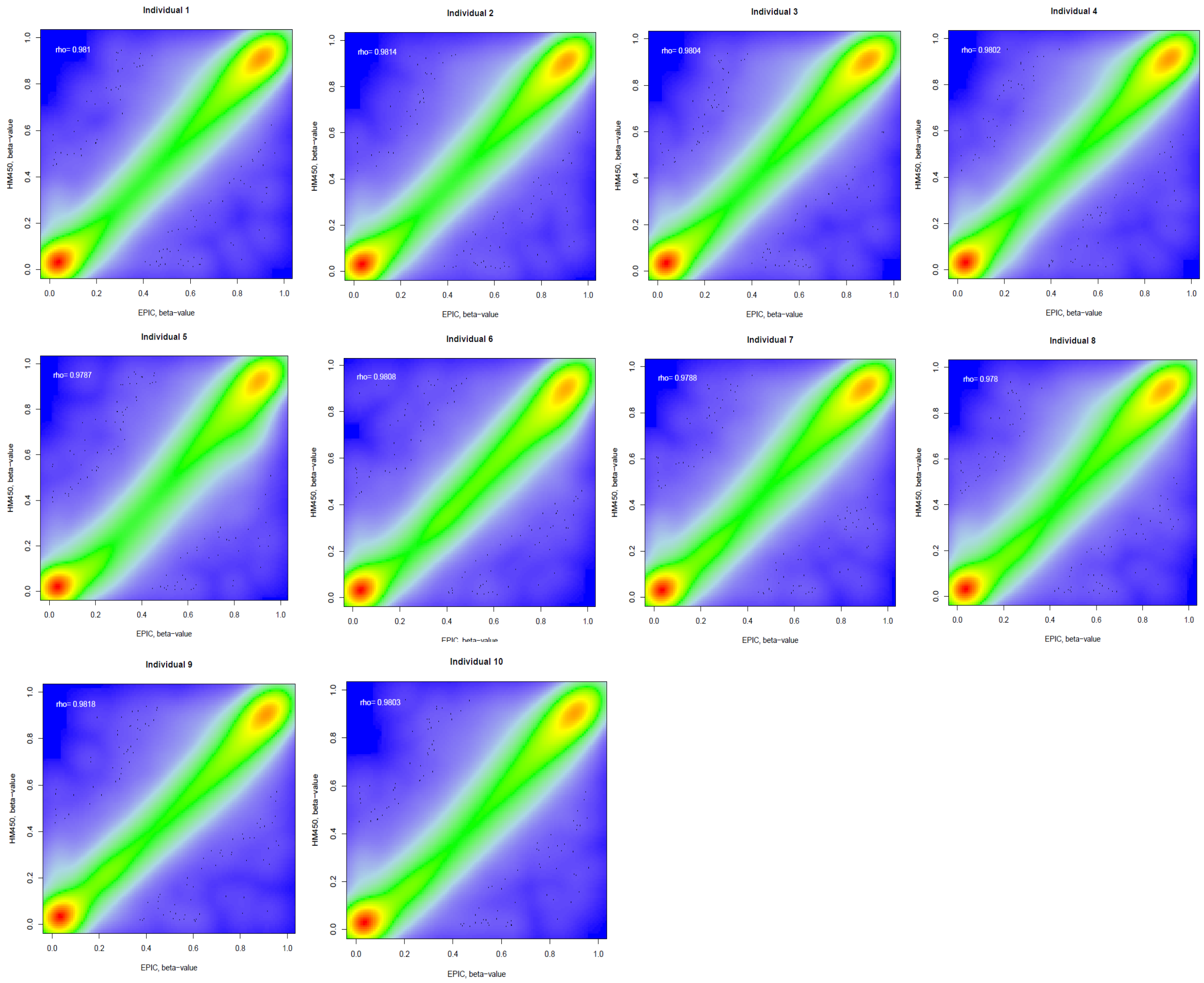

Figure S11: Quality control plot of bisulfite conversion.

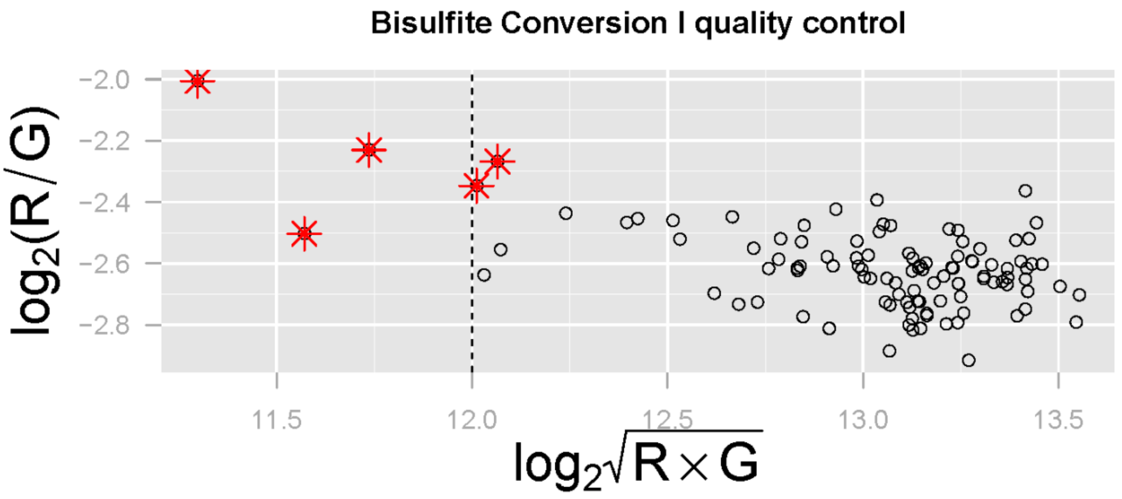

The performance of bisulfite conversion quality control probes is plotted for all DNA methylation samples. Red stars denote samples that failed on the basis of any of the five quality metrics. R=Red Channel. G=Green Channel.

Figure S12: Quality control plot of overall sample quality based on sample-dependent control probes (Non-Polymorphic quality control probes)

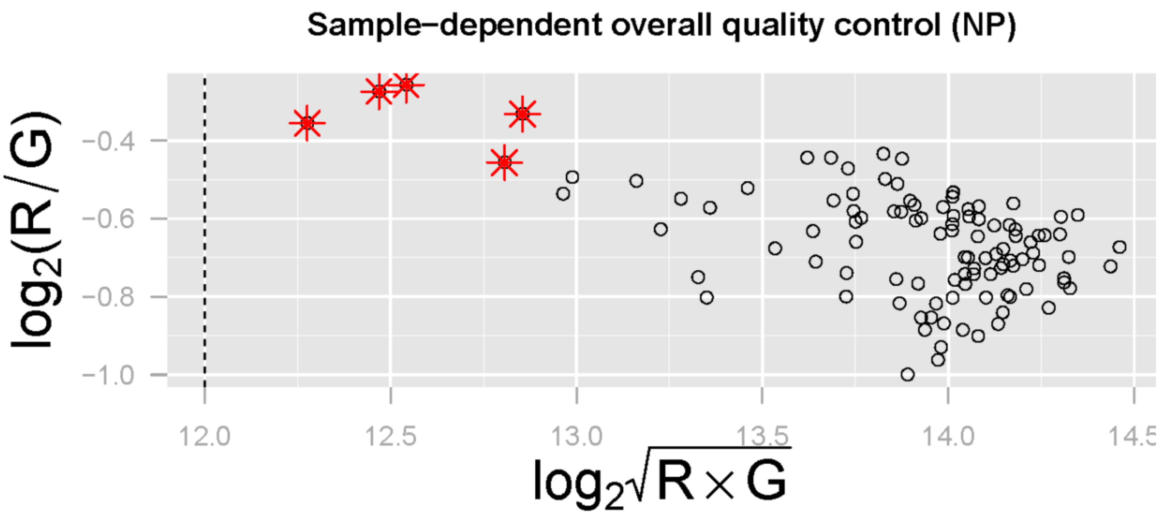

The performance of Non-Polymorphic quality control probes is plotted for all DNA methylation samples. Red stars denote samples that failed on the basis of any of the five quality metrics. R=Red Channel. G=Green Channel.

Figure S13: Quality control plot of the median Methylated versus Unmethylated signal intensity.

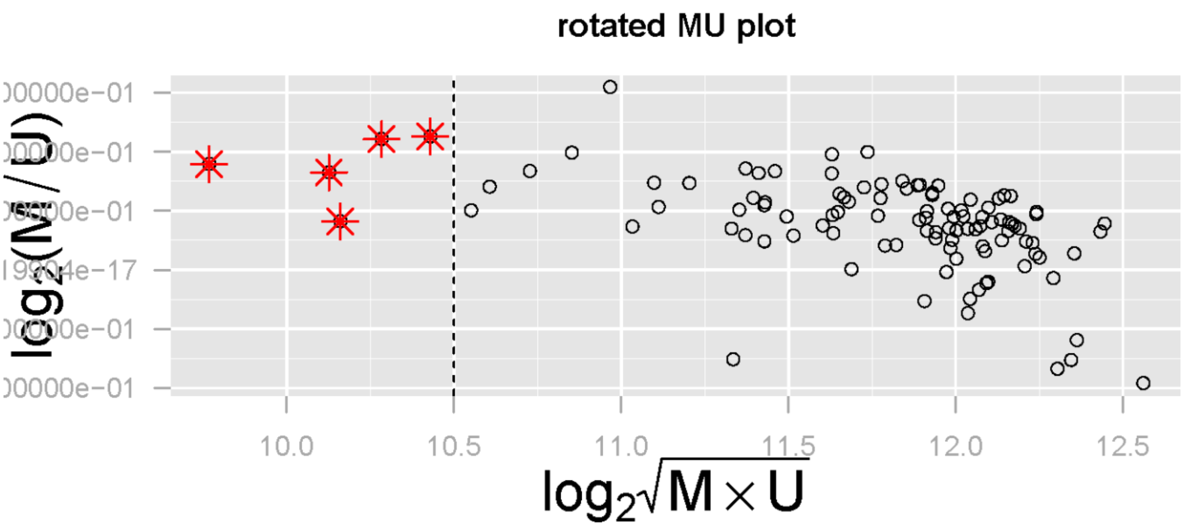

The relationship between the Median Methylated (M) and Unmethylated (U) signal intensity is plotted for all DNA methylation samples. Red stars denote samples that failed on the basis of any of the five quality metrics.

Figure S14: Quality control plot based on sample-independent hybridization control probes.

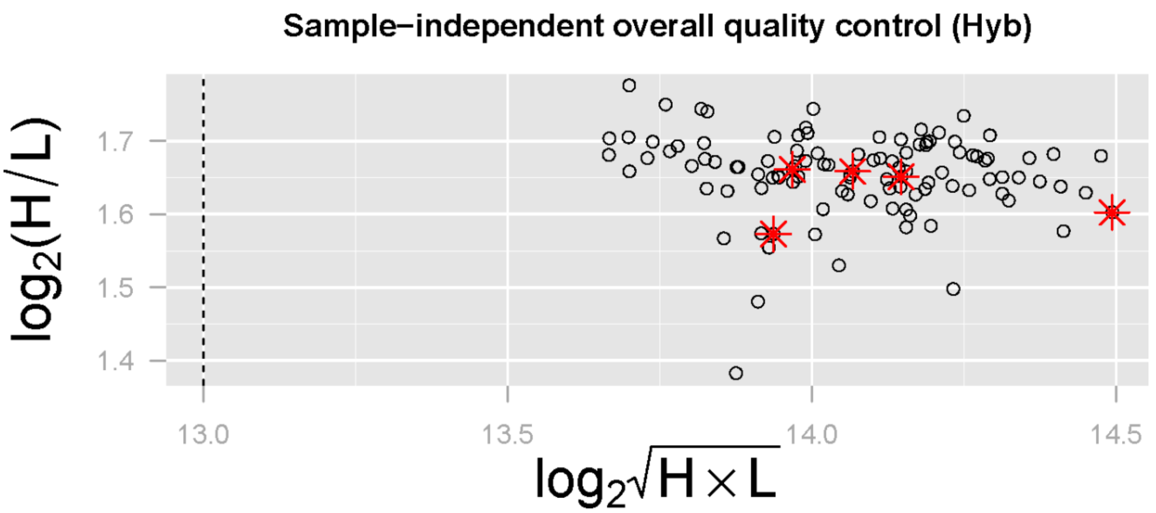

The performance of sample-independent hybridization control probes is plotted for all DNA methylation samples. Red stars denote samples that failed on the basis of any of the five quality metrics.

Figure S15: Quality control plot showing the proportion of probes with a detection p-value < 0.01 within samples.

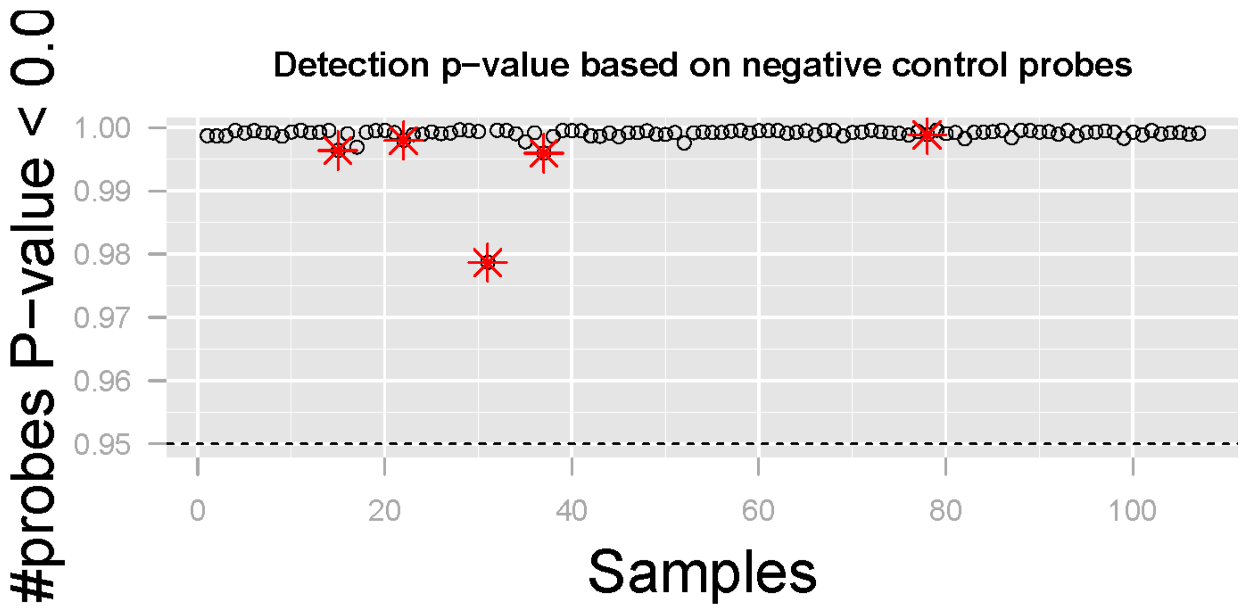

For all methylation samples, the proportion of probes per sample with a detection p-value < 0.01 is plotted (y-axis). The detection p-value indicates whether the probe signal exceeds the background signal, where the background signal is calculated using the negative control probes. Red stars denote samples that failed on the basis of any of the five quality metrics.

Figure S16: Scree plots of PCs based on control probes.

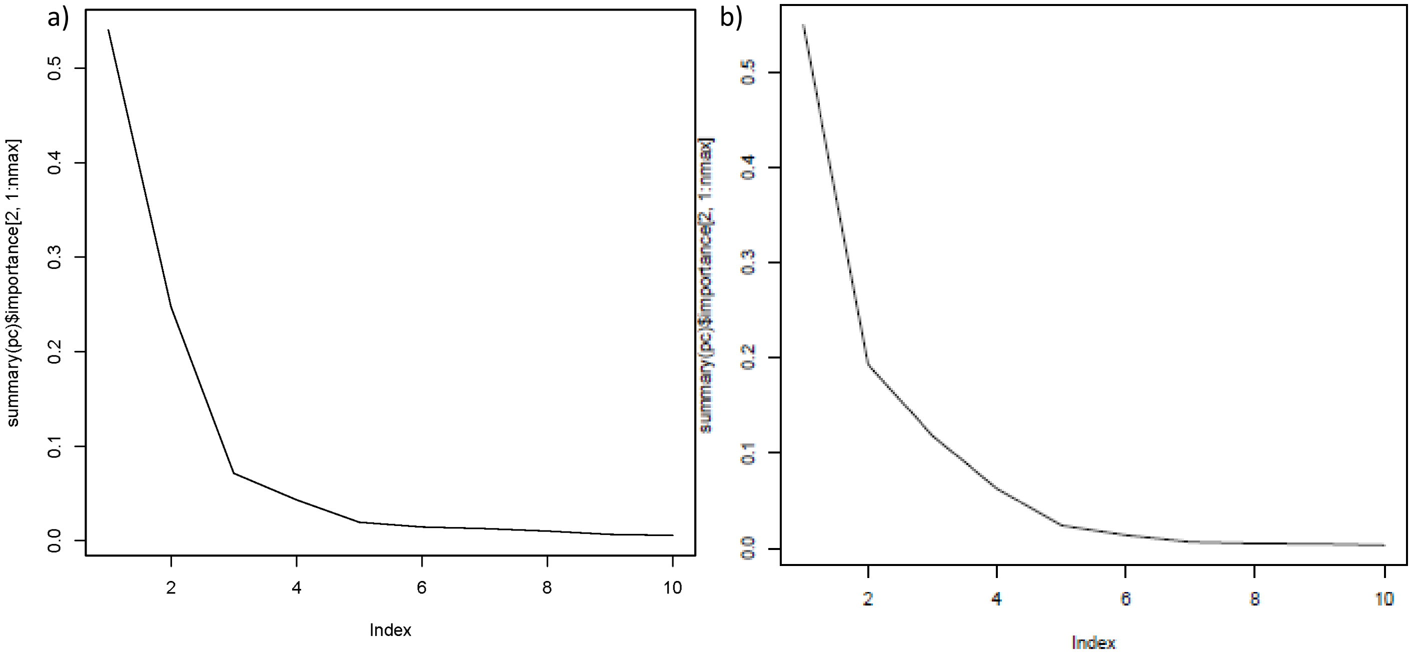

PC1 till PC10 are shown (x-axis). The y-axis shows the proportion of variance explained by each PC. a) EPIC arrays. b) merged dataset of EPIC and HM450 arrays.

Figure S17: Heatmap of the correlations of technical and biological variables with PCs based on the genome-wide methylation data prior to normalization

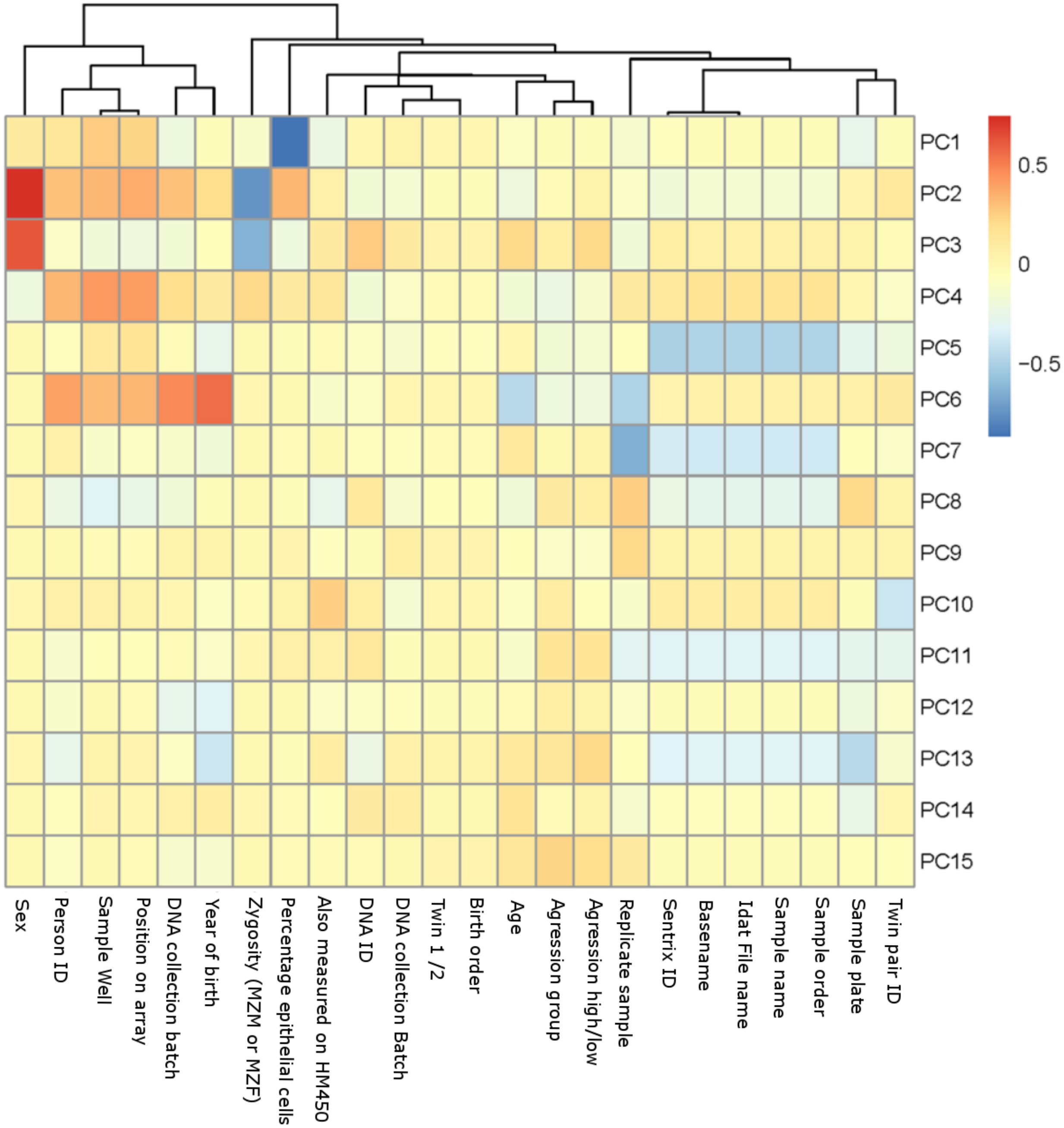

The legend indicates the correlation coefficient.

Figure S18: Heatmap of the correlations of technical and biological variables with PCs based on the genome-wide methylation data after functional normalization

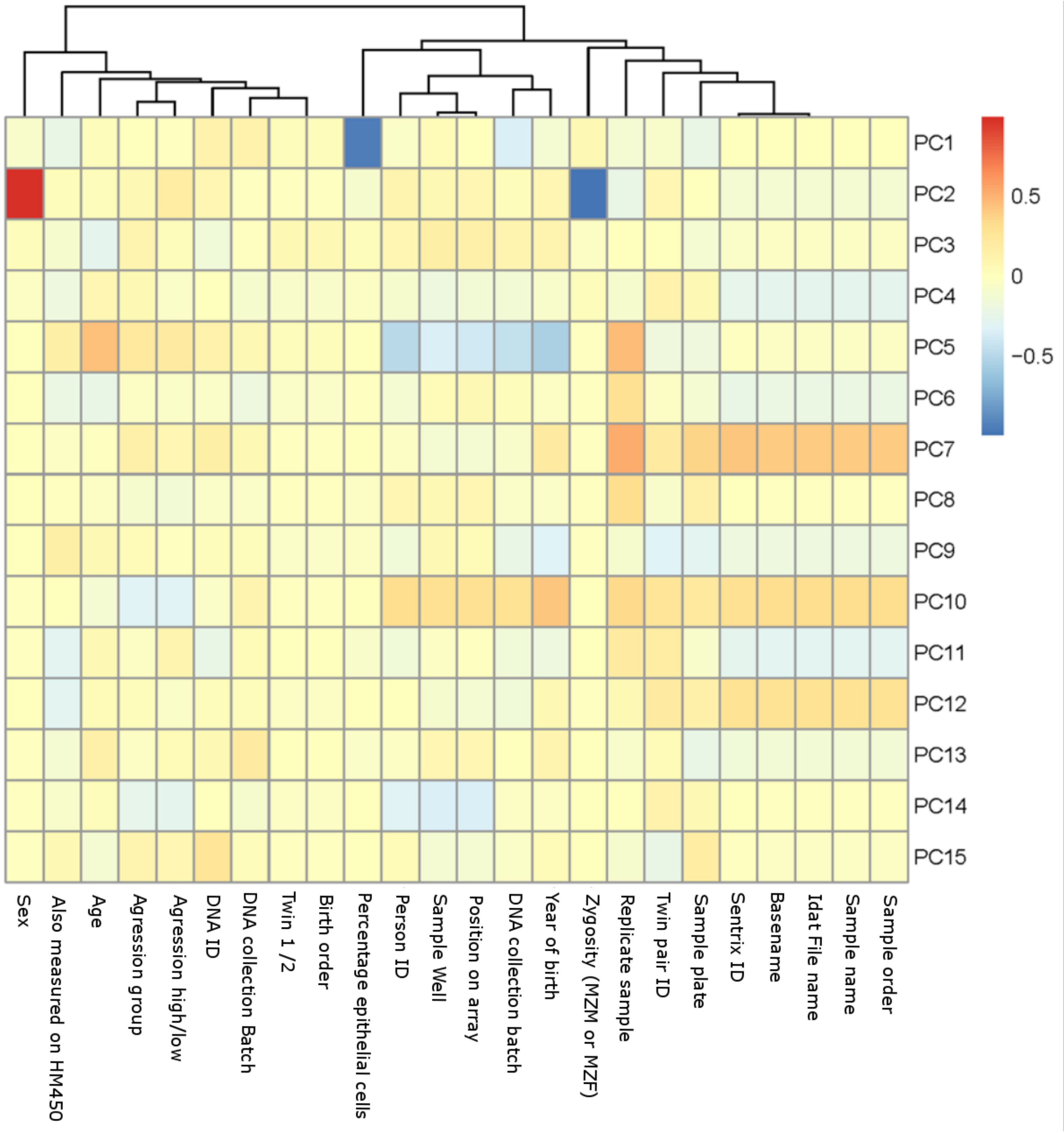

The legend indicates the correlation coefficient.

Figure S19: IBS mean-variance plot from omicsPrint of samples measured on EPIC

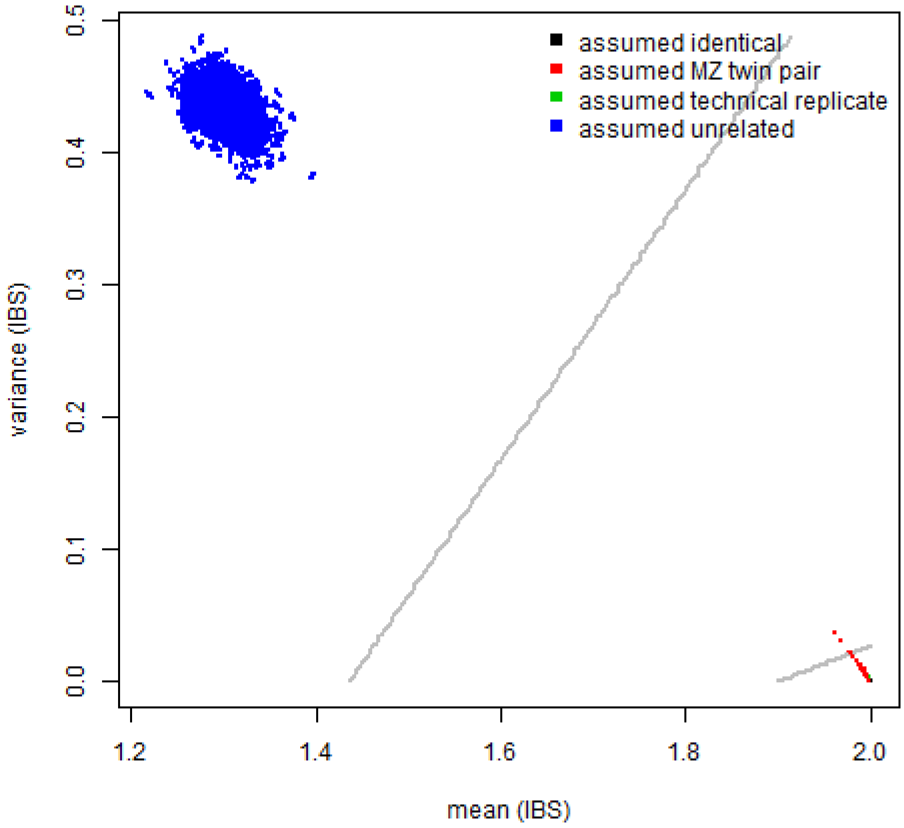

IBS was calculated based on 1156 SNPs interrogated from the EPIC data. The plot shows two clusters: samples from unrelated individuals (blue) and samples from MZ twin pairs and technical replicates of the same DNA (others). All pairwise comparisons between samples are plotted and identical denotes IBS sharing of a sample with itself.

Figure S17: IBS mean-variance plot from omicsPrint of matched samples measured on EPIC and HM450

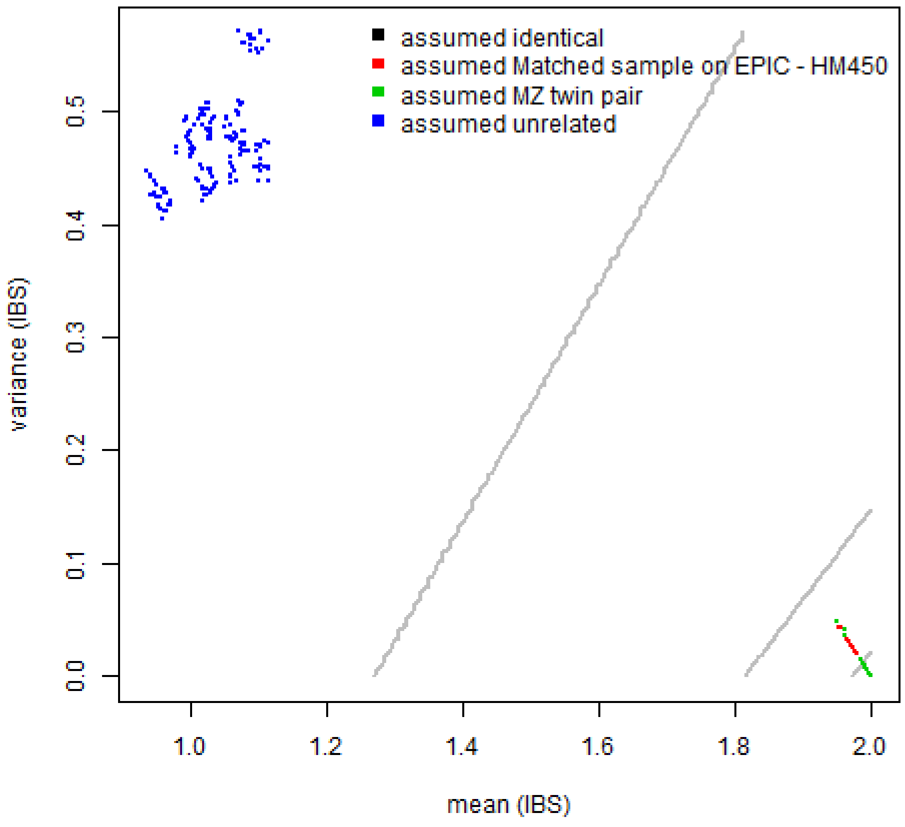

IBS was calculated based on 357 SNPs interrogated from the common probes of the combined EPIC-HM450 dataset. The plot shows two clusters: samples from unrelated individuals and samples from MZ twin pairs and measures of the same DNA on EPIC and HM450. All pairwise comparisons between samples are plotted and identical denotes IBS sharing of a sample with itself.
