# Supplementary figures and images for: Genome-wide analysis of DNA methylation in buccal cells: a study of monozygotic twins and mQTLs
Source: Epigenetics Chromatin. 2018 Sep 25;11:54. doi: 10.1186/s13072-018-0225-x (PMC6156977; doi:10.1186/s13072-018-0225-x)

DMPs in DNase I sites (probably TF sites) in cell lines for encode Unnamed

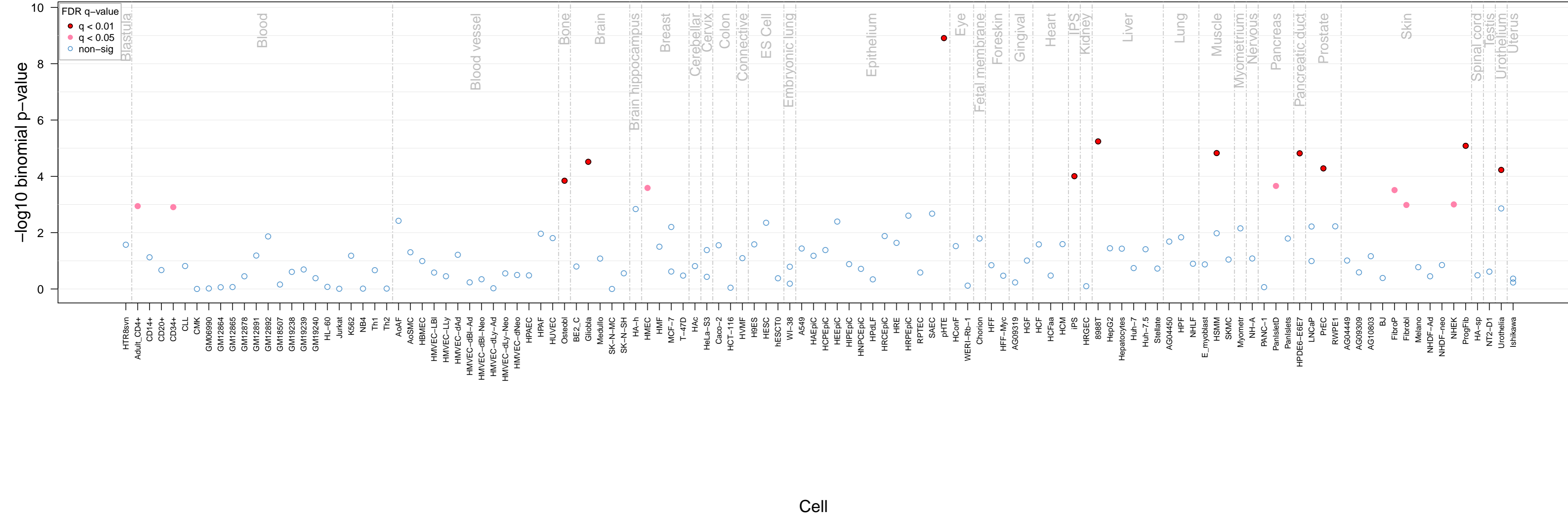

Supplement: Supplementary file 2 — Additional file 2. DHS enrichment for methylation sites with large MZ twin correlation, unadjusted for cellular composition. [file 13072_2018_225_MOESM2_ESM.pdf]

DMPs in DNase I sites (probably TF sites) in cell lines for encode Unnamed

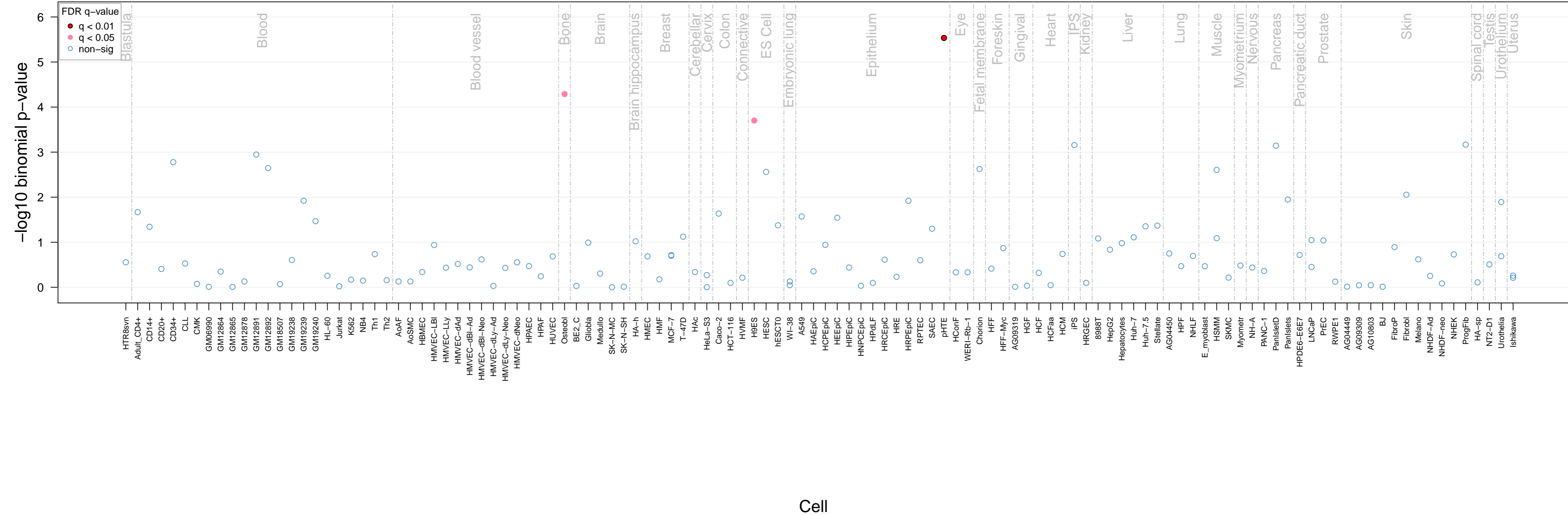

Supplement: Supplementary file 3 — Additional file 3. DHS enrichment for methylation sites with large MZ twin correlation, adjusted for cellular composition. [file 13072_2018_225_MOESM3_ESM.pdf]

DMPs in DNase I sites (probably TF sites) in cell lines for erc2–H3–all Unnamed

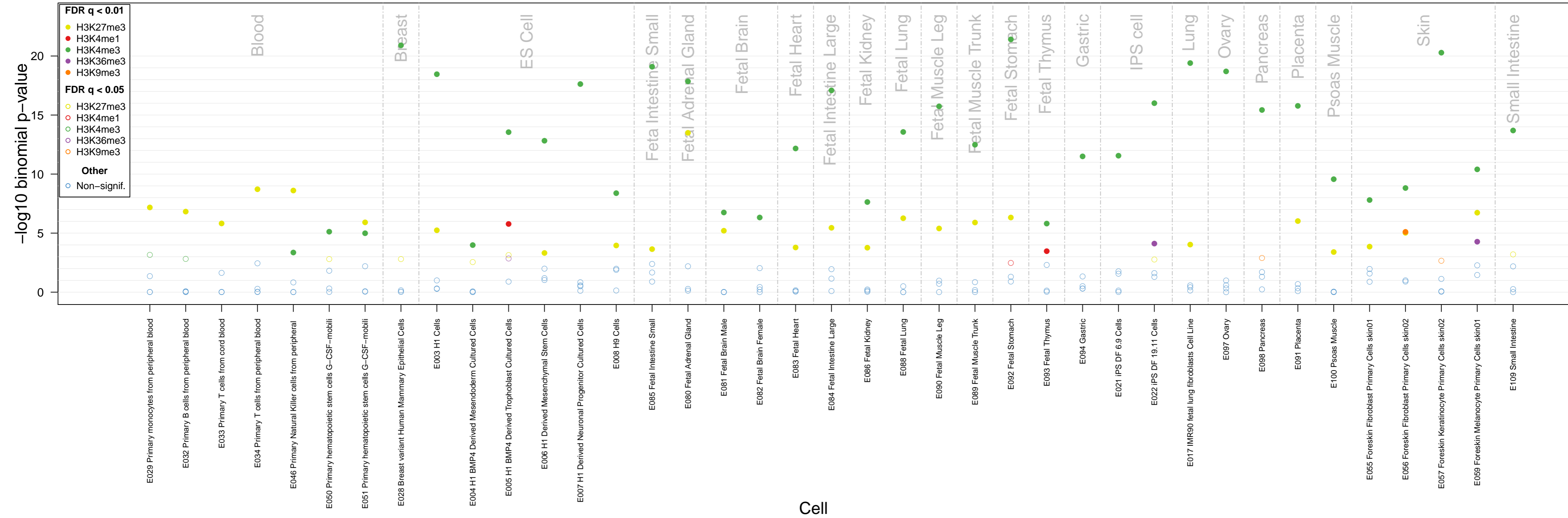

Supplement: Supplementary file 6 — Additional file 6. Histone H3 mark enrichment for methylation sites with large MZ twin correlation, unadjusted for cellular composition. [file 13072_2018_225_MOESM6_ESM.pdf]

DMPs in DNase I sites (probably TF sites) in cell lines for erc2–H3–all Unnamed

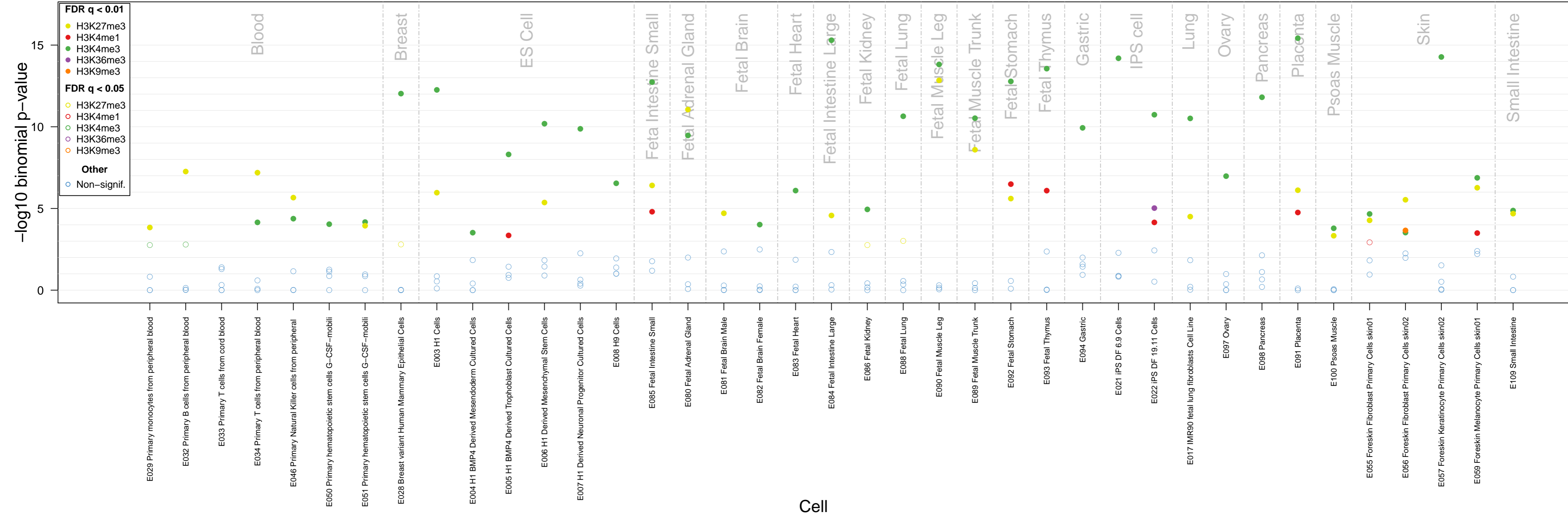

Supplement: Supplementary file 7 — Additional file 7. Histone H3 mark enrichment for methylation sites with large MZ twin correlation, adjusted for cellular composition. [file 13072_2018_225_MOESM7_ESM.pdf]

DMPs in DNase I sites (probably TF sites) in cell lines for encode Unnamed

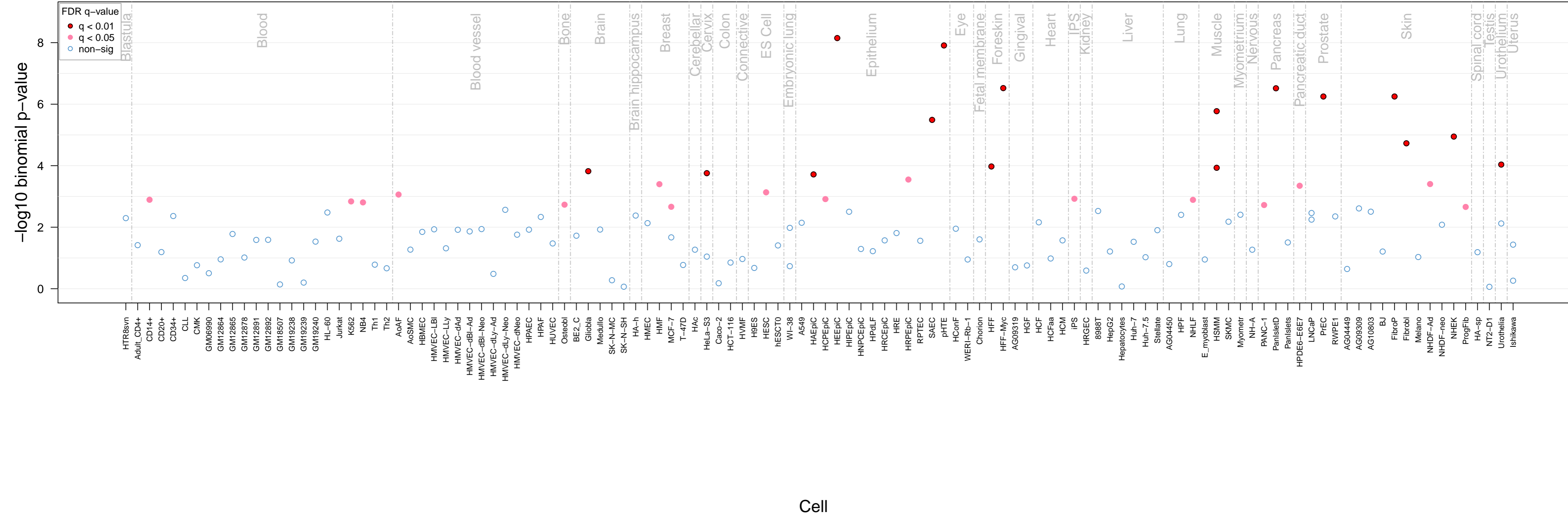

Supplement: Supplementary file 8 — Additional file 8. DHS enrichment for methylation sites with the strongest mQTLs. [file 13072_2018_225_MOESM8_ESM.pdf]

DMPs in DNase I sites (probably TF sites) in cell lines for erc2–H3–all Unnamed

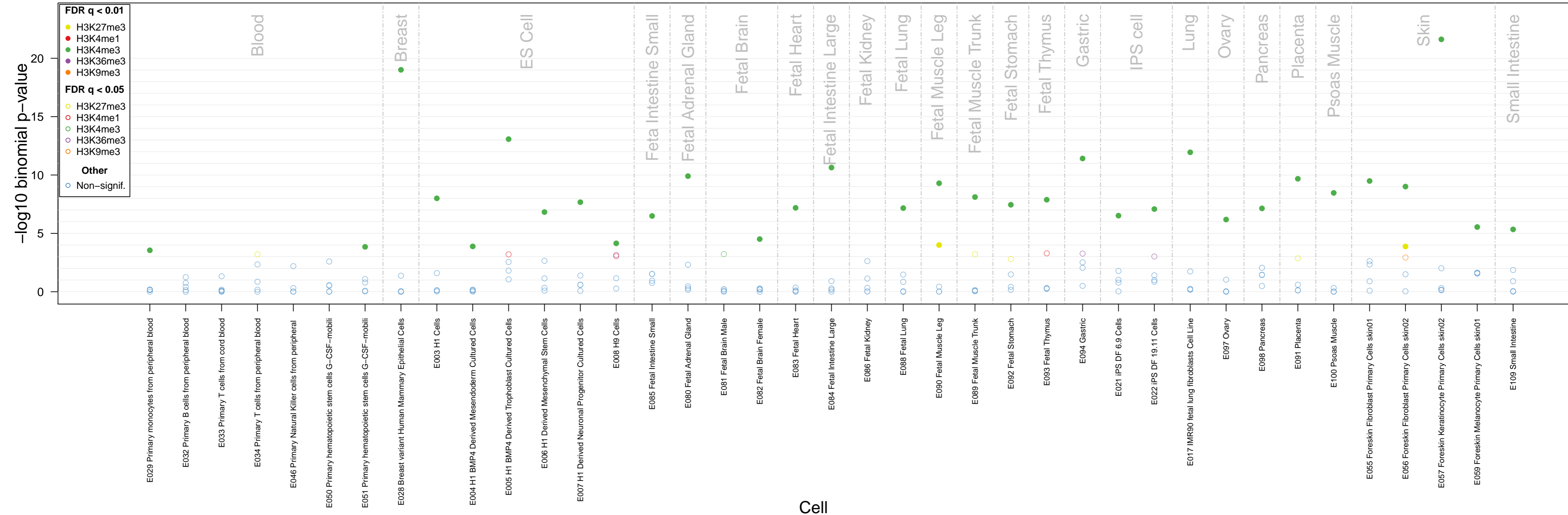

Supplement: Supplementary file 10 — Additional file 10. Histone H3 mark enrichment for methylation sites with the strongest mQTLs. [file 13072_2018_225_MOESM10_ESM.pdf]
