# Supplementary material for: Genome-wide analysis of DNA methylation in buccal cells: a study of monozygotic twins and mQTLs
Source: Epigenetics Chromatin. 2018 Sep 25;11:54. doi: 10.1186/s13072-018-0225-x (PMC6156977; doi:10.1186/s13072-018-0225-x)

DMPs in DNase I sites (probably TF sites) in cell lines for erc2−chromatin15state−all Unnamed

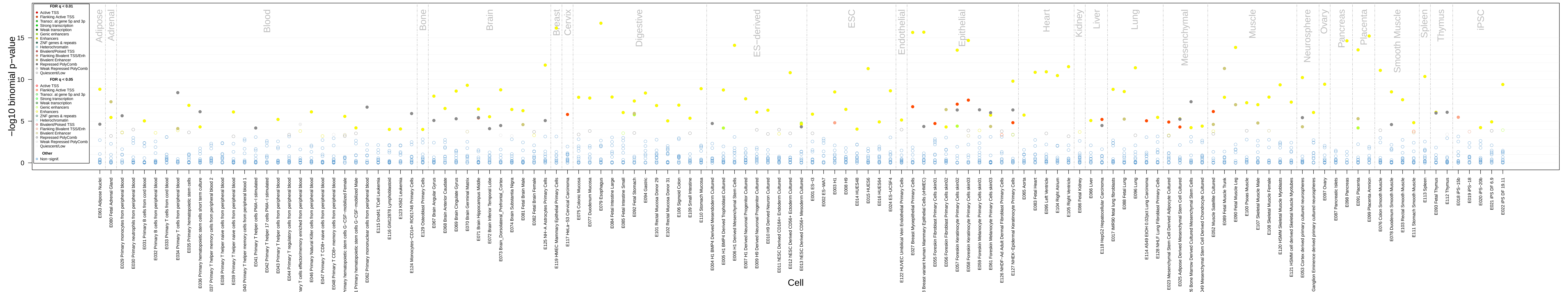

Supplement: Supplementary file 4 — Additional file 4. Chromatin state enrichment for methylation sites with large MZ twin correlation, unadjusted for cellular composition. [file 13072_2018_225_MOESM4_ESM.pdf]
